# Optimal Control of Hydrogen Atom-Like Systems as Thermodynamic Engines in Finite Time

**DOI:** 10.3390/e22101066

**Published:** 2020-09-23

**Authors:** Johann Christian Schön

**Affiliations:** Max Planck Institute for Solid State Research, Heisenbergstr. 1, D-70569 Stuttgart, Germany; C.Schoen@fkf.mpg.de

**Keywords:** optimal control, thermodynamic cycles, finite-time thermodynamics, thermodynamic length, hydrogen atom, nano-size engines, a-thermal cycle

## Abstract

Nano-size machines are moving from only being topics of basic research to becoming elements in the toolbox of engineers, and thus the issue of optimally controlling their work cycles becomes important. Here, we investigate hydrogen atom-like systems as working fluids in thermodynamic engines and their optimal control in minimizing entropy or excess heat production in finite-time processes. The electronic properties of the hydrogen atom-like system are controlled by a parameter κ reflecting changes in, e.g., the effective dielectric constant of the medium where the system is embedded. Several thermodynamic cycles consisting of combinations of iso-κ, isothermal, and adiabatic branches are studied, and a possible a-thermal cycle is discussed. Solving the optimal control problem, we show that the minimal thermodynamic length criterion of optimality for finite-time processes also applies to these cycles for general statistical mechanical systems that can be controlled by a parameter κ, and we derive an appropriate metric in probability distribution space. We show how the general formulas we have obtained for the thermodynamic length are simplified for the case of the hydrogen atom-like system, and compute the optimal distribution of process times for a two-state approximation of the hydrogen atom-like system.

## 1. Introduction

Optimal control of physical and chemical systems, and of the processes taking place in such systems, has been a major goal since the beginning of scientific investigations [1,2]. Essentially any application of scientific insights to practical problems has constituted such an effort in optimal control of some kind—even if not formulated as a mathematical control problem—, with the objective ranging from minimizing the difference of the values of characteristic parameters and quantities between the ideal theory and the real experiment [3,4], to maximizing the amount or quality of the desired output for given material and technical constraints. One can distinguish between those controls that are based on practical limitations due to the availability of tools and materials or lack thereof, and those based on the laws of physics.

Perhaps the classical examples of optimal control based on laws of physics have been the analyses of thermodynamic cycles [5,6,7], where the famous formulas for maximal efficiencies of hypothetical engines are sometimes given a status nearly equal to the fundamental laws of thermodynamics [6]. Such formulas have typically been derived under the assumption of infinite time available for each step of the cycles, allowing us to move from equilibrium state to equilibrium state. About fifty years ago, engineers and scientists began to question this assumption and re-formulated the optimal control problem by demanding that the cycle should be performed in a given finite amount of time, leading to the development of the field of finite-time thermodynamics [8,9,10,11].

A second aspect of optimal control based on the laws of physics is to reduce the engine under consideration to the most elementary physical systems that are stripped of all weaknesses and complications which are associated with the macroscopic aspects of the experimental apparatus employed in their realization, resulting in the creation and investigation of molecular machines [12]. Typically, this involves reducing the size of the system in the sense that we are dealing with a macroscopic system as a (non-interacting) ensemble of elementary but microscopic systems. Of course, in practice, isolating the individual elementary system often requires a very large experimental apparatus, but for the purpose of the analysis of the physics and the optimal control of the system, this counts as “environment” and “control tools”, whose optimality in themselves are usually irrelevant to the issue of optimally controlling the (ensemble of) elementary system(s) as such. Many experiments have been performed where single atoms or ions in vacuum [13,14,15] or single defects in solids [16,17] have been studied and controlled in some fashion.

A third feature of achieving optimal control of systems on the level of physical laws is dealing with the quantum nature of these elementary systems, which is forced upon us when reducing the elementary systems to atomic dimensions. While the quantum aspects are unavoidable, one can nevertheless often separate them from the issue of optimal control one investigates, especially if the time resolution of the control is so large that many quantum aspects can be captured by, e.g., effective decay rates.

From the point of view of a theorist, such a reduction to elementary physical systems is often a desirable feature because it allows us to focus on the elementary system itself—which can, in many cases, be analyzed analytically to a certain point—while assigning all other aspects to a generic environment. Examples are the optimal control of harmonic oscillator systems, which have been studied both on the classical and on the quantum mechanical level [18,19,20,21,22,23,24,25,26], spin systems [26,27,28], particles in a box [26], and generic two-level systems [29]. 

In this study, we are going to analyze the basic concepts of optimal control at the example of a hydrogen atom-like system, which we employ as an engine, or more specifically, the working fluid of such an engine, to perform a thermodynamic cycle within finite time. On the technical mathematical level, the electronic Hamiltonian possesses a very high degree of symmetry allowing at least some analytical treatment. On the level of possible applications, there exist systems equivalent to hydrogen-like atoms, e.g., excitons inside a solid [30], that can be controlled by modifying the environment while the hydrogen atom-like system remains in its state. Controlling such a system might allow us to construct nano-size engines inside solids. Alternatively, we can consider isolated hydrogen atoms inside a cavity [31], where also the conditions can be controlled such that the hydrogen atom becomes a modified hydrogen atom.

We will analyze the stages of the thermodynamic control cycle on the general level of probability distributions, both for the ideal (infinite-time) cycle and the cycle at finite time, derive an optimality criterion for minimal waste of work/minimal excess production of heat (dissipation)/minimal production of entropy during finite-time processes for general statistical mechanical systems, and apply these formulas to the example of the hydrogen atom-like system. Note that we are dealing with an “electronic” states-based engine, not with a standard thermal atom movement-based engine as studied, e.g., in [13].

## 2. Background

### 2.1. Aspects of the Hydrogen Atom-Like System

Due to the high symmetry of the Coulomb potential, the electronic Schrödinger equation for the hydrogen atom can be solved analytically, and we get for the energy eigenvalues

(1) $$E_{n}=-\frac{\alpha }{n^{2}}$$

where $\alpha =\frac{me^{4}}{2\hbar^{2}}$. The degeneracy of the n’th energy level is $g\left(n\right)=n^{2}$. In principle, we need to add a factor 2 for the spin degeneracy of the electron, but this factor is not relevant for this study and will be dropped.

If we consider hydrogen atom-like systems, the formula for the energy is modified by, e.g., changing the mass of the electron or quasi-particle $m\to m^{*}$, i.e., $\alpha \to \alpha^{*}=\frac{m^{*}}{m}\alpha$, by changing the effective charge that generates the Coulomb field $e\to q_{1}{}^{*}$ and/or the effective charge of the quasi-particle $e\to q_{2}{}^{*}$, i.e., $\alpha \to \alpha^{*}=\frac{m{\left(q_{2}{}^{*}\right)}^{2}{\left(q_{1}{}^{*}\right)}^{2}}{2\hbar^{2}}$ or by introducing a shielding dielectric constant ε≠1, such that $E_{n}=-\frac{\alpha }{n^{2}}$, or combinations thereof. Quite generally, we note that these modifications can be incorporated in a straightforward fashion by introducing a modification factor

(2) $$\kappa =\frac{m^{*}{\left(q_{2}{}^{*}\right)}^{2}{\left(q_{1}{}^{*}\right)}^{2}}{me^{4}\varepsilon^{2}},$$

such that

(3) $$E_{n}\left(\kappa \right)=-\kappa \frac{\alpha }{n^{2}}=\kappa E_{n}\left(\kappa =1\right).$$

**Figure 1:**
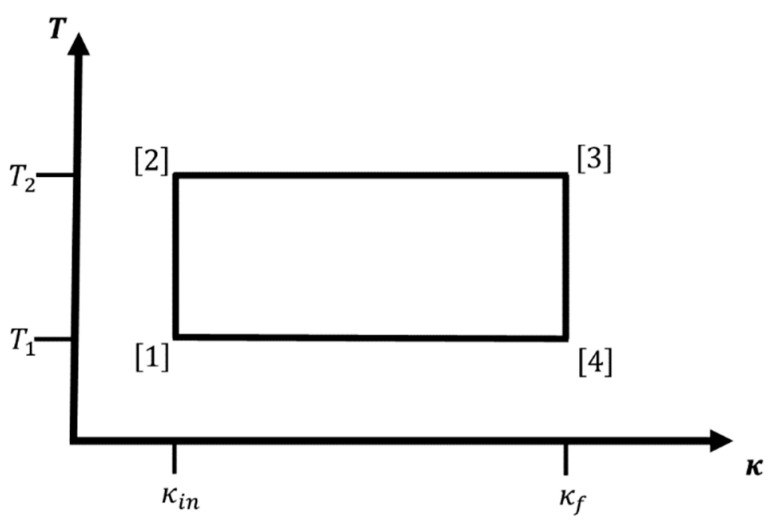
Sketch of an iso-κ-isothermal cycle for a hydrogen atom-like system. Branches [1]→[2] and [3]→[4] are iso-κ-legs and branches [2]→[3] and [4]→[1] are isothermal legs, respectively. The four corners of the cycle are the points $\left[1\right]=\left(\kappa^{\left[1\right]},T^{\left[1\right]}\right)=\left(\kappa_{in},T_{1}\right)$, $\left[2\right]=\left(\kappa^{\left[2\right]},T^{\left[2\right]}\right)=\left(\kappa_{in},T_{2}\right)$, $\left[3\right]=\left(\kappa^{\left[3\right]},T^{\left[3\right]}\right)=\left(\kappa_{f},T_{2}\right)$ and $\left[4\right]=\left(\kappa^{\left[4\right]},T^{\left[4\right]}\right)=\left(\kappa_{f},T_{1}\right)$ in the (κ,T) plane. Note that for given $\kappa_{in}$, $\kappa_{f}>\kappa_{in}$ and $T^{\left[1\right]}=T_{1}$, there remains only one variable, e.g., $T^{\left[2\right]}=T_{2}$, we are free to choose; all other variables are fixed by the choice of path types. All cycles for which $T_{2}>T_{1}$ are feasible iso-κ-isothermal cycles.

**Figure 2:**
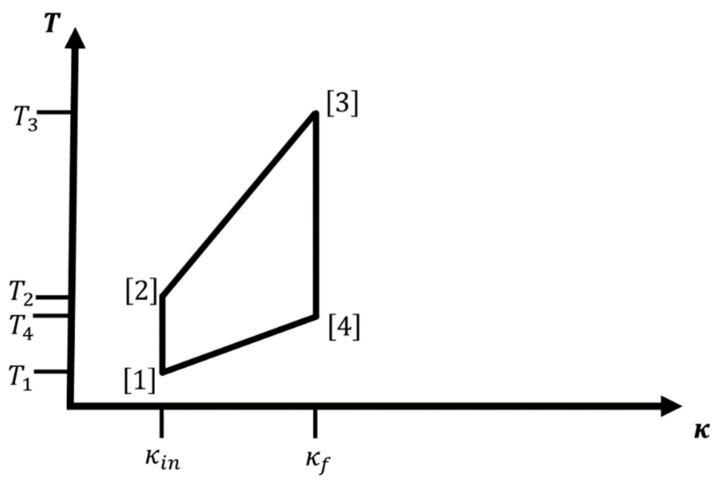
Sketch of an iso-κ-adiabatic cycle for a hydrogen atom-like system. Branches [1]→[2] and [3]→[4] are iso-κ-legs and branches [2]→[3] and [4]→[1] are (special) adiabatic legs, respectively. Note that the adiabatic legs run along straight lines through the origin. The four corners of the cycle are the points $\left[1\right]=\left(\kappa^{\left[1\right]},T^{\left[1\right]}\right)=\left(\kappa_{in},T_{1}\right)$, $\left[2\right]=\left(\kappa^{\left[2\right]},T^{\left[2\right]}\right)=\left(\kappa_{in},T_{2}\right)$, $\left[3\right]=\left(\kappa^{\left[3\right]},T^{\left[3\right]}\right)=\left(\kappa_{f},T_{3}\right)$, and $\left[4\right]=\left(\kappa^{\left[4\right]},T^{\left[4\right]}\right)=\left(\kappa_{f},T_{4}\right)$ in the (κ,T) plane. Note that for given $\kappa_{in}$, $\kappa_{f}>\kappa_{in}$, and $T^{\left[1\right]}=T_{1}$ there remains only one variable, e.g., $T^{\left[2\right]}=T_{2}$, we are free to choose; all other variables are fixed by the choice of path types. All cycles for which $T_{2}>T_{1}$ are feasible iso-κ-adiabatic cycles.

**Figure 3:**
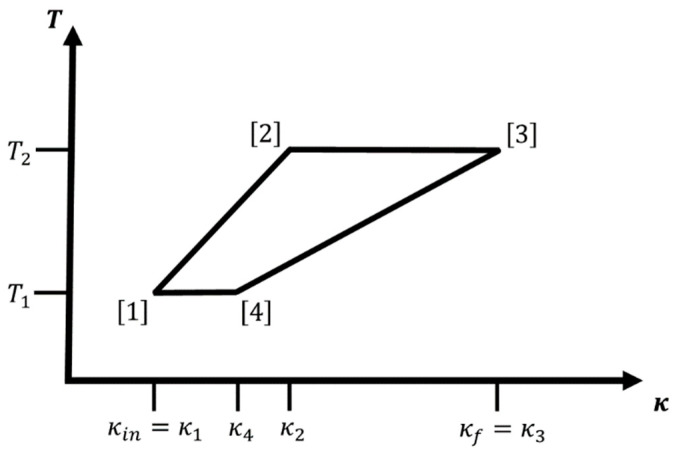
Sketch of an adiabatic-isothermal cycle for a hydrogen atom-like system. Branches [1]→[2] and [3]→[4] are (special) adiabatic legs and branches [2]→[3] and [4]→[1] are isothermal legs, respectively. Note that the adiabatic legs run along straight lines through the origin. The four corners of the cycle are the points $\left[1\right]=\left(\kappa^{\left[1\right]},T^{\left[1\right]}\right)=\left(\kappa_{in},T_{1}\right)$, $\left[2\right]=\left(\kappa^{\left[2\right]},T^{\left[2\right]}\right)=\left(\kappa_{2},T_{2}\right)$, $\left[3\right]=\left(\kappa^{\left[3\right]},T^{\left[3\right]}\right)=\left(\kappa_{f},T_{2}\right)$, and $\left[4\right]=\left(\kappa^{\left[4\right]},T^{\left[4\right]}\right)=\left(\kappa_{4},T_{1}\right)$ in the (κ,T) plane. Note that for given $\kappa_{in}$, $\kappa_{f}>\kappa_{in}$ and $T^{\left[1\right]}=T_{1}$, there remains only one variable, e.g., $T^{\left[2\right]}=T_{2}$, we are free to choose; all other variables are fixed by the choice of path types. However, only cycles for which $T_{2}^{max}>T_{2}>T_{1}$, with $\;T_{2}^{max}=\frac{\kappa_{f}\;}{\kappa_{in}}T_{1}$, are feasible adiabatic-isothermal cycles for the hydrogen atom-like system.

If we can apply external forces or fields to the system such that κ (i.e., $m^{*}$, $q_{1,2}{}^{*}$ and/or ε) can be smoothly varied, then we can modify the energy levels in a controlled fashion. In that case, the hydrogen atom-like system is used as the working fluid of an engine, where the changing of the energy levels leads to a change in energy that may be extracted as work. A change of the statistical occupation of the various energy levels at given temperature T is associated with the entropy content and the heat exchanges of the system, and we can define work cycles in, e.g., (κ,T)-space. Examples for individual legs (or branches) of such a cycle would be adiabatic or isothermal changes in κ from some initial value $\kappa_{in}$ to a final value $\kappa_{f}$, and iso-κ changes in temperature where we keep κ constant (see Figure 1, Figure 2 and Figure 3 below). 

In this context, one should note that, e.g., the dielectric constant ε=ε(p,T) depends on both temperature and pressure, complicating the issue considerably; for small ranges of temperature and pressure, ε(p,T) commonly changes with temperature and pressure in an approximately linear fashion in a solid [32,33], i.e., $\varepsilon \left(p,T\right)\approx \varepsilon \left(p_{ref},T_{ref}\right)+\gamma_{T}\left(p_{ref},T_{ref}\right)\left(T-T_{ref}\right)+\gamma_{p}\left(p_{ref},T_{ref}\right)\left(p-p_{ref}\right)$. Depending on the type of system, we may have $\gamma_{T}\left(p_{ref},T_{ref}\right)>0$ and $\gamma_{p}\left(p_{ref},T_{ref}\right)<0$, respectively [32], or the opposite [33], or other combinations. Thus, e.g., we find that $\kappa \sim \frac{1}{\varepsilon^{2}}$ increases with pressure and decreases with temperature for CdF_2_ [32], and we could translate our analysis from using (κ,T) to (p,T) as control variables, in principle.

Of course, all energy levels of the hydrogen atom-like system will exhibit nonzero occupancy in thermodynamic equilibrium in general—in principle, even including the continuous distribution of energy levels associated with modified plane–wave states beyond the ionization energy (although these will most likely be irrelevant due to the assumed localization or confinement of the electron). However, for practical calculations, one can often simplify the system by considering, e.g., only the two lowest energy levels $E_{1}$ and $E_{2}$. Frequently, such simplified models can yield some quantitative results and help to better understand the qualitative behavior of the full system [34]. Finally, we note that there are other ways to modify the electronic states of the hydrogen atom-like system, e.g., by breaking the spherical symmetry of the Hamiltonian, such that Equations (1)–(3) no longer apply. Since the corresponding changes in the eigenvalue spectrum of such a modified Hamiltonian are very specific to the way we break the symmetry and need to be treated on a case-by-case basis, we are not going to discuss such systems and only allow modifications of the hydrogen atom-like system that preserve the spherical symmetry and the 1/r dependence of the Coulomb potential. 

### 2.2. Thermodynamic Cycles for the Hydrogen Atom-Like System

In general, we can distinguish two ways to change the occupation of the energy levels: directly via radiation with suitable frequencies corresponding to energy differences between specific energy levels, and stochastically by contact to heat baths at various temperatures. However, in the case of a radiation driven a-thermal cycle, we are not dealing with a system close to thermodynamic equilibrium, and the classical thermodynamic optimal control might not be suitable. Thus, in this study, we focus on the more familiar cycles involving interactions with heat reservoirs. 

Regarding the notation, we write $X^{\left(j\right)}$ or $X_{j\to j+1}$ to indicate that the variable, parameter, or quantity X belongs to leg (j) which runs from corner [j] to corner [j+1] of the four-leg cycle (corner [5] = corner [1], of course). Furthermore, quantities associated with corner [j] are referred to as $X^{\left[j\right]}$. This notation is introduced to keep the labeling of legs and corners distinct from the one for quantities such as the probability distribution ${\vec{p}}_{i}$ at step i along each branch when analyzing the effect of finite-time processes. Finally, the subscripts {κ;T}, {κ;S}, and {S;T} indicate that the quantity is associated with an iso-κ-isothermal, iso-κ-adiabatic, or adiabatic-isothermal cycle, respectively.

If we allow interaction with a heat bath, we need to consider an ensemble of identical isolated localized hydrogen atom-like systems such that we can apply statistical mechanics to evaluate the (equilibrium) probabilities of occupation of the individual energy levels. From a statistical point of view, the microstates correspond to the eigenstates of the hydrogen-like atom. For concreteness, we always define (without loss of generality) $\kappa_{in}$ and $\kappa_{f}>\kappa_{in}$ to denote the smallest and largest value of κ on the cycle, and $T^{\left[1\right]}=T_{1}$ as the lowest temperature in the cycle. Starting point of the cycle is always $\left(\kappa^{\left[1\right]},T^{\left[1\right]}\right)=\left(\kappa_{in},T_{1}\right)$. Together with the assignment of path types to the legs (adiabatic, isothermal, iso-κ), there is only one additional temperature, which we can choose, e.g., $T^{\left[2\right]}=T_{2}$ or $T^{\left[4\right]}=T_{4}$; all other temperatures and κ values of the cycle are then fixed. We always assume smooth changes in both κ and T along the legs of the cycle, i.e., for example, the system is exposed to an infinite number of heat baths with intermediary temperatures when changing T. 

Note that we use the terms adiabatic and isentropic essentially interchangeably for thermodynamic equilibrium paths, since we assume that the system is in thermodynamic equilibrium both at the starting and at the end point of a leg. In the general case of such an adiabatic path, only the entropy remains constant along the path. However, for some physical systems, such as the hydrogen atom-like ones we are going to discuss, not only the entropy but also the equilibrium Boltzmann distribution remains unchanged along an adiabatic path, and we call such paths “special adiabatic”. 

For such a macroscopic system, Figure 1 and Figure 2 show cycles combining two iso-κ legs with two isothermal or adiabatic legs, respectively. We excite the ensemble of atoms via change in temperature $T^{\left[1\right]}\to T^{\left[2\right]}=T_{2}>T_{1}$, while we keep κ at the value $\kappa_{in}$: $\kappa^{\left[2\right]}=\kappa^{\left[1\right]}=\kappa_{in}$. Next, we perform/extract work while we increase κ from $\kappa^{\left[2\right]}=\kappa_{in}$ to $\kappa^{\left[3\right]}=\kappa_{f}>\kappa_{in}$. This can be done adiabatically, thus involving a concomitant change in temperature during the process ($T^{\left[2\right]}\to T^{\left[3\right]}=T_{3}>T_{2}$), or isothermally keeping the temperature constant at $T^{\left[2\right]}\to T^{\left[3\right]}=T_{3}=T_{2}$. In leg 3, we de-excite the atom ensemble via change in temperature $T^{\left[3\right]}\to T^{\left[4\right]}=T_{4}<T_{3}$, while we keep κ at the value $\kappa^{\left[3\right]}=\kappa^{\left[4\right]}=\kappa_{f}$, and finally, we decrease κ from $\kappa^{\left[4\right]}=\kappa_{f}$ back to $\kappa^{\left[1\right]}=\kappa_{in}$; again, this can be done either adiabatically ($T^{\left[4\right]}\to T^{\left[1\right]}=T_{1}<T_{4}$) or isothermally at $T^{\left[4\right]}\to T^{\left[1\right]}=T_{1}=T_{4}$. Note that for cycles containing two iso-κ branches, the only condition on $T_{2}$ is $T_{2}>T_{1}$. In the literature, one sometimes calls the iso-κ-adiabatic cycle an Otto cycle, in analogy to the isochore-adiabatic cycle, which is the underlying cycle of an Otto engine [7], and similarly, we could call the iso-κ-isothermal cycle a Stirling cycle in analogy to the isochore-isothermal cycle belonging to the Stirling engine [7].

An alternative type of cycle shown in Figure 3 would include no legs with constant κ; instead, we combine two adiabatic and two isothermal legs to the analogue of a Carnot cycle [7]. We adiabatically increase κ from $\kappa^{\left[1\right]}=\kappa_{in}$ to $\kappa^{\left[2\right]}=\kappa_{2}$, $\kappa_{in}<\kappa_{2}<\kappa_{f}$, appropriately changing the temperature during the process ($T^{\left[1\right]}\to T^{\left[2\right]}=T_{2}>T_{1}$), followed by an isothermal leg where we increase κ from $\kappa^{\left[2\right]}=\kappa_{2}$ to $\kappa^{\left[3\right]}=\kappa_{3}=\kappa_{f}$ while keeping the temperature constant at $T^{\left[2\right]}\to T^{\left[3\right]}=T_{3}=T_{2}$. Next, we adiabatically decrease κ from $\kappa^{\left[3\right]}=\kappa_{3}$ to $\kappa^{\left[4\right]}=\kappa_{4}$, $\kappa_{in}<\kappa_{4}<\kappa_{f}$, while appropriately changing the temperature ($T^{\left[3\right]}\to T^{\left[4\right]}=T_{4}<T_{3}$), and finally, we decrease κ from $\kappa^{\left[4\right]}=\kappa_{4}$ back to $\kappa^{\left[1\right]}=$ $\kappa_{in}$ while keeping the temperature constant $T^{\left[4\right]}\to T^{\left[1\right]}=T_{1}=T_{4}$.

Note that a feasible isothermal-adiabatic cycle usually will have an additional condition on $T^{\left[2\right]}$. For the special adiabatic legs of a hydrogen atom-like system, feasibility requires that the temperature at the second corner fulfills the condition

(4) $$T_{1}<T_{2}<T_{2}^{max},$$

where

(5) $$T_{2}^{max}=\frac{\kappa_{f}\;}{\kappa_{in}}T_{1};$$

otherwise, leg 3 would have a larger slope than leg 1, violating the condition that $\kappa_{in}<\kappa_{2},\kappa_{4}<\kappa_{f}$.

When analyzing the work performed and the heat exchanged along the legs of the cycles, we do not include the work done on the environment of the hydrogen atom-like system (e.g., the solid or the cavity), which is needed to vary κ. Of course, we can take that into account, e.g., via replacing κ by the pressure p as control variable if κ=κ(p) depends on pressure in a monotonic fashion, and then compute the work needed to establish such a pressure inside the solid. However, the focus is on the hydrogen-atom like system, and not on the whole solid or cavity where the system might be realized in the experiment. Thus, we will stay with using κ as the control variable, and only consider the change in the hydrogen atom-like system itself.

There are several general aspects that need to be considered regarding the equilibrium distributions, relaxation, and entropy/excess heat production along the cycles. How close are the instantaneous probability distributions over the states of the system to the (equilibrium) Boltzmann distributions at the various temperatures along its iso-κ legs? Similarly, for branches where κ is changed, how does the finite time available to vary κ affect our ability to keep the probability distribution close to the appropriate Boltzmann distribution for isothermal processes, and how close can the adiabatic path in (κ,T)-space which is realized in experiment, be to the ideal adiabatic path? All these questions directly lead to the issue of relaxation to the appropriate Boltzmann distributions in finite time, for which we can hope to derive general formulas as long as we can assume that the processes are slow enough for the system to always remain close to thermodynamic equilibrium throughout the cycle.

### 2.3. Statistical Mechanics and Thermodynamics of Cycles

Regarding the statistical mechanics and thermodynamics of cycles, the starting point of our analysis will be the first law of thermodynamics,

(6) dE=δQ−δW

along a piece of path in thermodynamic space, which is equivalent to δW=δQ−dE. Here, dE>0 means that the internal energy of the system is increased along the leg, and δQ>0 means that heat is added to the atom from the reservoir(s) the atom is in contact with along the path, thus increasing the system’s internal energy. δW>0 means that the system does work on the apparatus, radiation field or the environment in general, along the leg, and reduces the system’s internal energy in the process. To avoid confusion, we note that in the literature δW(>0) is frequently defined in an alternative fashion to refer to the amount of work done by the apparatus on the system increasing its internal energy; as a consequence, one then would write the first law as dE=δQ+δW and δW=dE−δQ.

The connection to statistical mechanics appears via the information theoretic definition of the entropy

(7) $$S=-k_{B}\sum_{j=1}^{N_{S}}p_{j}ln\left(p_{j}\right),$$

where $p_{j}$ is the probability for the microstate j to be occupied, and the sum is over all $N_{S}$ microstates [5]. In equilibrium at given temperature T, these probabilities $\pi_{j}$ correspond to the equilibrium Boltzmann probability distribution,

(8) $$\pi_{j}\left(T\right)=\frac{exp\left(-\frac{E_{j}}{k_{B}T}\right)}{Z\left(T\right)}$$

for the case of the canonical ensemble, where the sum over states $Z\left(T\right)=\sum_{k=1}^{N_{S}}exp\left(-\frac{E_{k}}{k_{B}T}\right)$ serves as the normalization of the probability distribution, and $E_{j}$ is the energy of microstate j. Keep in mind that the equilibrium distribution maximizes the entropy, for a given set of boundary conditions that are, e.g., defining the ensemble. 

If we consider the energy levels $E_{n}\left(={\tilde{E}}_{n}\right)$ of the states instead of the microstates themselves in the formula of the entropy, then we need to include the degeneracies $g_{n}$ of the energies ${\tilde{E}}_{n}$. As far as the equilibrium probabilities are concerned, we can assume that the probabilities of occupying two states i and j with the same energy $E_{i}=E_{j}={\tilde{E}}_{n}$ are equal; for convenience, we define $\rho_{n}=\pi_{i}=\pi_{j}$, and thus

(9) $${\tilde{\pi }}_{n}=g_{n}\rho_{n},$$

is the equilibrium probability to find the system with energy ${\tilde{E}}_{n}$. In general, we can write the equilibrium entropy as

(10) $$S=-k_{B}\sum_{n=1}^{N_{E}}g_{n}\rho_{n}ln\left(\rho_{n}\right)=-k_{B}\sum_{n=1}^{N_{E}}{\tilde{\pi }}_{n}ln\left(\rho_{n}\right)=-k_{B}\sum_{n=1}^{N_{E}}{\tilde{\pi }}_{n}ln\left(\frac{{\tilde{\pi }}_{n}}{g_{n}}\right)=\;-k_{B}\sum_{n=1}^{N_{E}}{\tilde{\pi }}_{n}\left[ln\left({\tilde{\pi }}_{n}\right)-ln\left(g_{n}\right)\right],$$

where now the summation goes over the $N_{E}$ energy levels and ${\tilde{\pi }}_{n}$ are the equilibrium probabilities to find the system in a state with energy ${\tilde{E}}_{n}$. Note that for non-degenerate states, i.e., $g_{n}=1$, this expression reverts to the original microstate-based formula, and for the special case ${\tilde{\pi }}_{n}=1$ for n=m while ${\tilde{\pi }}_{n}=0$ for n≠m, we obtain the formula for the entropy in the microcanonical ensemble, $S=k_{B}ln\left(g_{m}\right)$. 

Finally, for those probability distributions where we deviate from the equilibrium distribution but nevertheless can assume that the probabilities of being in a state with energy $E_{i}=E_{j}={\tilde{E}}_{n}$ are the same, we can define $r_{n}=p_{i}=p_{j}$, and thus ${\tilde{p}}_{n}=g_{n}r_{n}$. Then, we get for the entropy the expression $S=-k_{B}\sum_{n=1}^{N_{E}}g_{n}r_{n}ln\left(r_{n}\right)=-k_{B}\sum_{n=1}^{N_{E}}{\tilde{p}}_{n}ln\left(\frac{{\tilde{p}}_{n}}{g_{n}}\right)$ and the normalization $\sum_{n=1}^{N_{E}}g_{n}r_{n}=\sum_{n=1}^{N_{E}}{\tilde{p}}_{n}=\sum_{j=1}^{N_{S}}p_{j}=1$. Note that this is a substantial restriction in the set of allowed probability distributions; thus, unless this assumption is valid, we need to employ the microstate formulation of the problem.

As a shorthand notation, we can represent the probability distributions by vectors $\vec{p}=\left(p_{1},\ldots ,p_{N_{S}}\right)$, and expressions like $\sum_{j=1}^{N_{S}}p_{j}ln\left(p_{j}\right)$ can be written as

(11) $$\sum_{j=1}^{N_{S}}p_{j}ln\left(p_{j}\right)=\vec{p}\cdot ln\left(\vec{p}\right),$$

where $ln\left(\vec{p}\right)=\left(ln\left(p_{1}\right),\ldots ,ln\left(p_{N_{S}}\right)\right)$. Similarly, $\vec{E}=\left(E_{1},\ldots ,E_{N_{S}}\right)$, and we can relate the equilibrium probabilities of the Boltzmann distribution to the energies via

(12) $$\vec{E}=-k_{B}Tln\left(\vec{\pi }\right)+C\left(\vec{\pi }\right)\vec{1}=-k_{B}Tln\left(\vec{\pi }\right)-k_{B}Tln\left(g\left(\vec{\pi }\right)\right)\vec{1},$$

where $g\left(\vec{\pi }\right)$ is a constant that can be related to an overall shift of the energy scale, and $\vec{1}=\left(1,\ldots ,1\right)$ [35]. The expectation value of the energy is then given by

(13) $$\sum_{j=1}^{N_{S}}p_{j}E_{j}=\vec{E}\cdot \vec{p},$$

or, for the case of the expectation value in equilibrium, $\overset{N_{S}}{\underset{j=1}{\sum }}\pi_{j}E_{j}=\vec{E}\cdot \vec{\pi }$. Note that some of these energies will appear many times in this vector if the energy level is degenerate. Another example is the expectation value of the square of the energy, $\overset{N_{S}}{\underset{j=1}{\sum }}\pi_{j}{\left(E_{j}\right)}^{2}=\vec{E^{2}}\cdot \vec{\pi }$, where $\vec{E^{2}}=\left({\left(E_{1}\right)}^{2},\ldots ,{\left(E_{N_{S}}\right)}^{2}\right)$. If appropriate, we can replace the sums over the $N_{S}$ microstates by sums over the $N_{E}$ energies, i.e., $\overset{N_{S}}{\underset{j=1}{\sum }}\pi_{j}E_{j}=\overset{N_{E}}{\underset{j=1}{\sum }}{\tilde{\pi }}_{j}{\tilde{E}}_{j}=\vec{\tilde{E}}\cdot \vec{\tilde{\pi }}$, where $\vec{\tilde{\pi }}=\left({\tilde{\pi }}_{1},\ldots ,{\tilde{\pi }}_{N_{E}}\right)$ and $\vec{\tilde{E}}=\left({\tilde{E}}_{1},\ldots ,{\tilde{E}}_{N_{E}}\right)$, or for the entropy $S=-k_{B}\overset{N_{E}}{\underset{n=1}{\sum }}{\tilde{\pi }}_{n}ln\left(\rho_{n}\right)=-k_{B}\vec{\tilde{\pi }}\cdot ln\left(\vec{\rho }\right)$ where $ln\left(\vec{\rho }\right)=\left(ln\left(\rho_{1}\right),\ldots ,ln\left(\rho_{N_{E}}\right)\right)=\left(ln\left(\frac{{\tilde{\pi }}_{1}}{g_{1}}\right),\ldots ,ln\left(\frac{{\tilde{\pi }}_{N_{E}}}{g_{N_{E}}}\right)\right)$. In the case of the single atom-like system, the microstates correspond to the eigenfunctions of the Hamiltonian of an electron (or a quasi-particle) in a (shielded) Coulomb potential. Note that we are not considering the degeneracy due to the multiple copies of the atom-like system in the ensemble—the ensemble is only introduced to allow us to visualize the occupation probabilities of the single atom-like system with which we are dealing.

Since the energy is a state function, the change of its equilibrium expectation value

(14) $$\langle E\rangle =\sum_{j=1}^{N_{S}}E_{j}\pi_{j}=\vec{E}\cdot \vec{\pi }$$

along a leg (j) of the path does not depend on the choice of path, i.e., the total change in energy can be computed directly from taking the difference between the two states,

(15) $${\left(\Delta E\right)}_{j\to j+1}={\langle E\rangle }^{\left[j+1\right]}-{\langle E\rangle }^{\left[j\right]}.$$

For the heat delivered/absorbed by the atom along a leg (j) (parametrized by $\lambda \in \left[\lambda^{\left[j\right]},\lambda^{\left[j+1\right]}\right]$), we can use the formula

(16) $$Q_{\vec{\pi }}^{\left(j\right)}=\int_{S^{\left[j\right]}}^{S^{\left[j+1\right]}}TdS=-k_{B}\int_{\lambda^{\left[j\right]}}^{\lambda^{\left[j+1\right]}}T\left(\lambda \right)\frac{d}{d\lambda }\left[\vec{\pi }\cdot ln\left(\vec{\pi }\right)\right]d\lambda =-k_{B}\int_{\lambda^{\left[j\right]}}^{\lambda^{\left[j+1\right]}}T\left(\lambda \right)\left[ln\left(\vec{\pi }\left(\lambda \right)\right)+\vec{1}\right]\cdot \;\frac{d\vec{\pi }}{d\lambda }d\lambda =-k_{B}\int_{{\vec{\pi }}^{\left[j\right]}}^{{\vec{\pi }}^{\left[j+1\right]}}T\left(\vec{\pi }\right)\left[ln\left(\vec{\pi }\right)+\vec{1}\right]\cdot d\vec{\pi },$$

if we stay at the equilibrium distribution along the path, and an analogous expression in $\vec{p}$ if we deviate from the equilibrium distribution because of, e.g., finite-time effects:

(17) $$Q_{\vec{p}}^{\left(j\right)}=\int_{S^{\left[j\right]}}^{S^{\left[j+1\right]}}TdS=-k_{B}\int_{\lambda^{\left[j\right]}}^{\lambda^{\left[j+1\right]}}T\left(\lambda \right)\left[ln\left(\vec{p}\left(\lambda \right)\right)+\vec{1}\right]\cdot \frac{d\vec{p}}{d\lambda }d\lambda .$$

Finally, the work performed by the atom can be computed via the first law by taking the difference between δQ and dE along the path. Along the legs where κ is constant, both Q and ΔE will vary as they are both functions of the probability distribution, which in turn evolves as the temperature changes. To compute ΔE is no problem, but the expression for Q is nontrivial. We can switch coordinates to follow the change in temperature instead of the change in entropy,

(18) $$Q_{j\to j+1}=\int_{S^{\left[j\right]}}^{S^{\left[j+1\right]}}TdS=\left[T^{\left[j+1\right]}S^{\left[j+1\right]}-T^{\left[j\right]}S^{\left[j\right]}\right]-\int_{T^{\left[j\right]}}^{T^{\left[j+1\right]}}SdT=\left[T^{\left[j+1\right]}S^{\left[j+1\right]}-T^{\left[j\right]}S^{\left[j\right]}\right]+\;k_{B}\int_{T^{\left[j\right]}}^{T^{\left[j+1\right]}}\vec{\pi }\left(T\right)\cdot ln\left(\vec{\pi }\left(T\right)\right)dT,$$

However, we note that this way of writing $Q^{\left(j\right)}$ does not necessarily make the integral easier to perform in general either, even though κ, and thus all the energy levels $E_{n}\left(\kappa \right)$, are constant along these legs of the cycle. However, since the work δW would usually be associated with changes in κ, we expect that W=0 along thermodynamic equilibrium paths with fixed κ, and therefore, Q=ΔE. Using Equations (16) and (12), we find:

(19) $$\begin{array}{cc} & W^{\left(j\right)}=Q^{\left(j\right)}-{\left(\Delta E\right)}^{\left(j\right)}=\int_{S^{\left[j\right]}}^{S^{\left[j+1\right]}}TdS-\left[{\langle E\rangle }^{\left[j+1\right]}-{\langle E\rangle }^{\left[j\right]}\right]\\ & =-\int_{\vec{\pi }\left(T^{\left[j\right]}\right)}^{\vec{\pi }\left(T^{\left[j+1\right]}\right)}k_{B}T\left[ln\left(\vec{\pi }\right)+\vec{1}\right]\cdot d\vec{\pi }-\vec{E}\left(\kappa_{in}\right)\cdot \left(\vec{\pi }\left(\kappa_{in},T^{\left[j+1\right]}\right)-\vec{\pi }\left(\kappa_{in},T^{\left[j\right]}\right)\right)\\ & =-\int_{\vec{\pi }\left(T^{\left[j\right]}\right)}^{\vec{\pi }\left(T^{\left[j+1\right]}\right)}k_{B}T\left[ln\left(\vec{\pi }\right)+\vec{1}\right]\cdot d\vec{\pi }-\int_{\vec{\pi }\left(T^{\left[j\right]}\right)}^{\vec{\pi }\left(T^{\left[j+1\right]}\right)}\vec{E}\left(\kappa_{in}\right)\cdot d\vec{\pi }\\ & =-\int_{\vec{\pi }\left(T^{\left[j\right]}\right)}^{\vec{\pi }\left(T^{\left[j+1\right]}\right)}\left\{k_{B}T\left[ln\left(\vec{\pi }\right)+\vec{1}\right]+\vec{E}\left(\kappa_{in}\right)\right\}\cdot d\vec{\pi }\\ & =-\int_{\vec{\pi }\left(T^{\left[j\right]}\right)}^{\vec{\pi }\left(T^{\left[j+1\right]}\right)}\left\{k_{B}T\left[ln\left(\vec{\pi }\right)+\vec{1}\right]-k_{B}T\left[ln\left(\vec{\pi }\right)+ln\left(g\left(\vec{\pi }\right)\right)\vec{1}\right]\right\}\cdot d\vec{\pi }\\ & =-\int_{\vec{\pi }\left(T^{\left[j\right]}\right)}^{\vec{\pi }\left(T^{\left[j+1\right]}\right)}\left\{k_{B}T\left[ln\left(\vec{\pi }\right)+\vec{1}-ln\left(\vec{\pi }\right)-ln\left(g\left(\vec{\pi }\right)\right)\vec{1}\right]\right\}\cdot d\vec{\pi }\\ & =-\int_{\vec{\pi }\left(T^{\left[j\right]}\right)}^{\vec{\pi }\left(T^{\left[j+1\right]}\right)}\left\{k_{B}T\left[1-ln\left(g\left(\vec{\pi }\right)\right)\right]\right\}\vec{1}\cdot d\vec{\pi }=0\;, \end{array}$$

since $\vec{1}\cdot d\vec{\pi }=0$ along a thermodynamic equilibrium path because of probability conservation. Note that we have not exploited the fact that we are dealing with a hydrogen atom-like system, and thus this result is general for any statistical mechanical system along legs with κ = constant, even for the most general case of many control parameters $\kappa \hat{=}\vec{\kappa }=\left(\kappa_{a},\kappa_{b},\ldots \right)$.

If we consider the complete cycle, the changes in energy will sum to zero, and thus W=Q over the whole cycle, i.e., the net heat added to the atom over the cycle must be converted into net work done by the atom on the apparatus. The discussion in this subsection is standard procedure from equilibrium thermodynamics [5,6], but has been included to establish the concepts and notation needed for analyzing the thermodynamics of the cycles of statistical mechanical systems in finite time in the next sections.

## 3. Optimal Control Criterion for Finite-Time Thermodynamic Cycles of General Statistical Mechanical Systems

### 3.1. Finite-Time Optimal Control along a General Path in (κ,T) Space

If thermodynamic processes are performed in finite time, one usually encounters deviations in the heat exchanged and work performed along the path from those values that one finds for the ideal quasi-static processes for which infinite times are available, because the system cannot maintain thermodynamic equilibrium over the whole cycle when the total time τ is finite. As a consequence, a net amount of entropy is produced over the whole cycle or excess heat/work is found while the system tries to move towards or stay close to the elusive thermal equilibrium along the path. Typically, one would attempt to minimize this entropy or excess heat production, leading to the formulation of the minimum excess heat/work production optimal control problems discussed in finite-time thermodynamics. In this study, we will focus on minimum entropy production and minimum excess heat production; other possible optimal control objectives such as maximum power or maximum efficiency are not considered. In the following, the subscript Q will indicate minimization of excess heat, while the subscript S indicates minimization of entropy production.

In general, one would need to analyze each process in depth, in order to compute the excess heat or excess work associated with the given physical situation. However, we can perform an approximate analysis of the difference $I_{Q}=Q_{\vec{\pi }}-Q_{\vec{p}}$ between the heat $Q_{\vec{\pi }}$ we would obtain under the assumption that along the path the system is everywhere in thermodynamic equilibrium, i.e., ${\vec{p}}_{i}\left(T_{i}\right)={\vec{\pi }}_{i}\left(T_{i}\right)$, and the corresponding expression $Q_{\vec{p}}$ along the path for the real probability distribution where ${\vec{p}}_{i}\left(T_{i}\right)\neq {\vec{\pi }}_{i}\left(T_{i}\right)$, yet ${\vec{p}}_{i}\left(T_{i}\right)$ is nevertheless close to ${\vec{\pi }}_{i}\left(T_{i}\right)$ and just a bit "behind", i.e., we can approximate ${\vec{p}}_{i}\left(T_{i}\right)$ as

(20) $${\vec{p}}_{i}\left(T_{i}\right)={\vec{\pi }}_{i-1}\left(T_{i-1}\right),$$

For the remainder of this study, we will assume that Equation (20) is a suitable approximation; the issue of incomplete relaxation to equilibrium of ${\vec{p}}_{i}\left(T_{i}\right)$ to ${\vec{\pi }}_{i-1}\left(T_{i-1}\right)$ is discussed in Refs. [35,36].

Using Equations (16) and (17) where λ now corresponds to time, we get

(21) $$I_{Q}=Q_{\vec{\pi }}-Q_{\vec{p}}=-k_{B}\int_{0}^{\tau }T\left(t\right)\left\{\dot{\vec{\pi }}\cdot \left[ln\left(\vec{\pi }\left(t\right)\right)+\vec{1}\right]-\dot{\vec{p}}\cdot \left[ln\left(\vec{p}\left(t\right)\right)+\vec{1}\right]\right\}dt\approx -k_{B}\int_{0}^{\tau }T\left(t\right)\dot{\vec{\pi }}\cdot \;\left\{ln\left(\vec{\pi }\left(t\right)\right)-ln\left(\vec{p}\left(t\right)\right)\right\}dt\approx k_{B}\int_{0}^{\tau }T\left(t\right)\dot{\vec{\pi }}\cdot \left\{\frac{d}{d\vec{\pi }}ln\left(\vec{\pi }\left(t\right)\right)\cdot \left(\vec{\pi }\left(t\right)-\vec{p}\left(t\right)\right)\right\}dt\approx \;\sum_{i=1}^{N_{st}}\left({\vec{\pi }}_{i}-{\vec{\pi }}_{i-1}\right)\cdot \left\{\frac{k_{B}T_{i}}{{\overset{↔}{\pi }}_{i}}\right\}\left({\vec{\pi }}_{i}-{\vec{p}}_{i}\right)\approx \sum_{i=1}^{N_{st}}\left({\vec{\pi }}_{i}-{\vec{\pi }}_{i-1}\right)\cdot \left\{\frac{k_{B}T_{i}}{{\overset{↔}{\pi }}_{i}}\right\}\left({\vec{\pi }}_{i}-{\vec{\pi }}_{i-1}\right).$$

Here, $N_{st}$ is the number of possible steps in (κ,T) space during the finite time τ assuming that each temperature-plus-κ change requires a “relaxation-to-equilibrium” time of ${\left(\Delta t\right)}_{st}$, i.e., $\tau =N_{st}{\left(\Delta t\right)}_{st}$. In the above derivation, several approximations were applied. First, we have employed the fact that $\vec{p}\left(t\right)$ essentially follows $\vec{\pi }\left(t\right)$, and thus $\dot{\vec{\pi }}=\dot{\vec{p}}$ to first order in $\left(\vec{\pi }\left(t\right)-\vec{p}\left(t\right)\right)$. Furthermore, we assumed that $\left(\vec{\pi }\left(t\right)-\vec{p}\left(t\right)\right)$ is very small, and thus we can make a Taylor expansion of $ln\left(\vec{p}\left(t\right)\right)$ about $\vec{\pi }\left(t\right)$. Next, we assumed that we can make a large number of changes in (κ,T) along the path, such that we can replace the integral by a sum over such discrete changes, resulting in $\dot{\vec{\pi }}dt\approx \dot{\vec{\pi }}{\left(\Delta t\right)}_{st}\approx \left({\vec{\pi }}_{i}-{\vec{\pi }}_{i-1}\right)$, and finally we assumed that Equation (20) holds, i.e., ${\vec{p}}_{i}\approx {\vec{\pi }}_{i-1}$.

Analogously, we can derive an expression for the entropy production over the path as

(22) $$I_{S}=-k_{B}\int_{0}^{\tau }\left\{\dot{\vec{\pi }}\cdot \left[ln\left(\vec{\pi }\left(t\right)\right)+\vec{1}\right]-\dot{\vec{p}}\cdot \left[ln\left(\vec{p}\left(t\right)\right)+\vec{1}\right]\right\}dt\;\approx \sum_{i=1}^{N_{st}}\left({\vec{\pi }}_{i}-{\vec{\pi }}_{i-1}\right)\cdot \left\{\frac{k_{B}}{{\overset{↔}{\pi }}_{i}}\right\}\left({\vec{\pi }}_{i}-{\vec{\pi }}_{i-1}\right).$$

Looking at the expressions for the excess heat and the entropy production that are to be minimized, we see that ${\overset{↔}{M}}_{Q}=\left\{\frac{k_{B}T_{i}}{{\overset{↔}{\pi }}_{i}}\right\}$ and ${\overset{↔}{M}}_{S}=\left\{\frac{k_{B}}{{\overset{↔}{\pi }}_{i}}\right\}$, repectively, are positive definite diagonal matrices that can serve as metrics in the space of probability distributions with components

(23) $${\left({\overset{↔}{M}}_{Q}\right)}_{kl}={\left\{\frac{k_{B}T_{i}}{{\overset{↔}{\pi }}_{i}}\right\}}_{kl}=\frac{k_{B}T_{i}}{\pi_{k}\left(\kappa_{i},T_{i}\right)}\delta_{kl}\;\mathrm{and}\;{\left({\overset{↔}{M}}_{S}\right)}_{kl}={\left\{\frac{k_{B}}{{\overset{↔}{\pi }}_{i}}\right\}}_{kl}=\frac{k_{B}}{\pi_{k}\left(\kappa_{i},T_{i}\right)}\delta_{kl},$$

which allows us to define distances in thermodynamic space, and thus yield the corresponding thermodynamic lengths of the path. Note that both the temperature $T_{i}$ and ${\vec{\pi }}_{i}$ (which is a function of $\left(\kappa_{i},T_{i}\right))$ depend on i, and thus the metrics ${\overset{↔}{M}}_{Q/S}$ vary along the path. $I_{Q/S}$ is now minimized by picking the stopping points along the path such that the thermodynamic lengths

(24) $${\left(\Delta L_{Q}\right)}_{i}={\left[\left({\vec{\pi }}_{i}-{\vec{\pi }}_{i-1}\right)\left\{\frac{k_{B}T_{i}}{{\overset{↔}{\pi }}_{i}}\right\}\left({\vec{\pi }}_{i}-{\vec{\pi }}_{i-1}\right)\right]}^{1/2}\;\mathrm{or}\;{\left(\Delta L_{S}\right)}_{i}={\left[\left({\vec{\pi }}_{i}-{\vec{\pi }}_{i-1}\right)\left\{\frac{k_{B}}{{\overset{↔}{\pi }}_{i}}\right\}\left({\vec{\pi }}_{i}-{\vec{\pi }}_{i-1}\right)\right]}^{1/2},$$

respectively, of all $N_{st}$ pieces of the path are equal. This follows from the Cauchy–Schwarz inequality

(25) $$I_{Q/S}=\sum_{i=1}^{N_{st}}{\left[{\left(\Delta L_{Q/S}\right)}_{i}\right]}^{2}\geq \frac{1}{N_{st}}{\left[\sum_{i=1}^{N_{st}}{\left(\Delta L_{Q/S}\right)}_{i}\right]}^{2}=\frac{{\left[{\left(\Delta L_{Q/S}\right)}_{total}\right]}^{2}}{N_{st}},$$

implying that equality holds only if the lengths of all individual pieces ${\left(\Delta L_{Q/S}\right)}_{i}$ are equal,

(26) $${\left(\Delta L_{Q/S}\right)}_{i}=\frac{{\left(\Delta L_{Q/S}\right)}_{total}}{N_{st}}.$$

Note that this criterion of equal thermodynamic lengths of sub-pieces is equivalent to the constant thermodynamic speed criterion [37] employed, e.g., in the context of optimization of global optimization algorithms [38], where thermodynamic speed corresponds to thermodynamic distance per relaxation, i.e., time is measured in units of relaxation-to-equilibrium times. 

Since ${\overset{↔}{M}}_{Q}=\left\{\frac{k_{B}T_{i}}{{\overset{↔}{\pi }}_{i}}\right\}$ is a diagonal matrix, we can rewrite each term in the sum as

(27) $$\left({\vec{\pi }}_{i}-{\vec{\pi }}_{i-1}\right)\cdot \left\{\frac{k_{B}T_{i}}{{\overset{↔}{\pi }}_{i}}\right\}\left({\vec{\pi }}_{i}-{\vec{\pi }}_{i-1}\right)=k_{B}T_{i}\sum_{k=1}^{N_{S}}\frac{{\left(d\pi_{i}\right)}_{k}{\left(d\pi_{i}\right)}_{k}}{{\left(\pi_{i}\right)}_{k}},$$

where $d{\vec{\pi }}_{i}=\left({\vec{\pi }}_{i}-{\vec{\pi }}_{i-1}\right)=\left({\left(d\pi_{i}\right)}_{1},\ldots {\left(d\pi_{i}\right)}_{N_{S}}\right)$ is the infinitesimal difference between the equilibrium probability distributions at steps i−1 and i. For the total length of the path along, e.g., leg (j) with $N^{\left(j\right)}$ steps, we thus get the expression

(28) $$L_{Q}^{\left(j\right)}=\sum_{i=1}^{N^{\left(j\right)}}\sqrt{\left({\vec{\pi }}_{i}-{\vec{\pi }}_{i-1}\right)\cdot \left\{\frac{k_{B}T_{i}}{{\overset{↔}{\pi }}_{i}}\right\}\left({\vec{\pi }}_{i}-{\vec{\pi }}_{i-1}\right)}=\sum_{i=1}^{N^{\left(j\right)}}\sqrt{\left[k_{B}T_{i}\sum_{k=1}^{N_{S}}\frac{{\left(d\pi_{i}\right)}_{k}{\left(d\pi_{i}\right)}_{k}}{{\left(\pi_{i}\right)}_{k}}\right]}\approx \;\left|\int_{leg\;\left(j\right)}^{}\sqrt{\left[k_{B}T\left(\lambda \right)\sum_{k=1}^{N_{S}}\frac{{\left(d\pi \left(\lambda \right)\right)}_{k}{\left(d\pi \left(\lambda \right)\right)}_{k}}{{\left(d\lambda \right)}^{2}{\left(\pi \left(\lambda \right)\right)}_{k}}\right]}d\lambda \right|=\left|\int_{leg\;\left(j\right)}^{}\sqrt{k_{B}T\left(\lambda \right)}\sqrt{\left[\sum_{k=1}^{N_{S}}\frac{1}{{\left(\pi \left(\lambda \right)\right)}_{k}}{\left(\frac{{\left(d\pi \left(\lambda \right)\right)}_{k}}{d\lambda }\right)}^{2}\right]}d\lambda \right|,$$

where λ is the variable of integration along the branch (j). Note that we have taken the absolute value of the integral, since $L_{Q}^{\left(j\right)}\geq 0$ by definition, independent of whether λ increases or decreases along the path. Analogously, we obtain for $L_{S}^{\left(j\right)}$ the expression

(29) $$L_{S}^{\left(j\right)}=\left|\int_{leg\;\left(j\right)}^{}\sqrt{k_{B}}\sqrt{\left[\sum_{k=1}^{N_{S}}\frac{1}{{\left(\pi \left(\lambda \right)\right)}_{k}}{\left(\frac{{\left(d\pi \left(\lambda \right)\right)}_{k}}{d\lambda }\right)}^{2}\right]}d\lambda \right|.$$

Of course, we must first choose the path in (κ,T)-space between the starting and end point of the leg in such a way that its total length is minimal. For general statistical mechanical systems, where the Hamiltonian is controlled not only by one single parameter κ but by a whole set of such parameters $\vec{\kappa }=\left(\kappa_{a},\kappa_{b},\ldots \right)$, this task is usually very complex. However, for the individual legs of the cycles for the hydrogen atom-like system, this is trivial, as we have only one parameter which can be varied (either just κ with fixed T for the isothermal leg, or both κ and T but in a synchronized fashion along the adiabatic leg, or finally only T with fixed κ along the iso-κ leg), and thus the path itself is given, and we can only vary the location of the steps i along the path. One additional aspect needs to be addressed: the optimization encompasses the whole cycle, and thus the available time τ needs to be distributed over the four legs, i.e., we have $\tau =\tau^{\left(1\right)}+\tau^{\left(2\right)}+\tau^{\left(3\right)}+\tau^{\left(4\right)}$. In principle, this poses no difficulty as long as the relaxation times per step are the same for all legs, $\Delta t^{\left(j\right)}={\left(\Delta t\right)}_{T}={\left(\Delta t\right)}_{\kappa }={\left(\Delta t\right)}_{a}$, regardless of whether we are dealing with an isothermal, iso-κ, or adiabatic leg: knowing the thermodynamic lengths for the four legs $L^{\left(1\right)}$, $L^{\left(2\right)}$, $L^{\left(3\right)}$, and $L^{\left(4\right)}$, we use $L_{cycle}=L^{\left(1\right)}+L^{\left(2\right)}+L^{\left(3\right)}+L^{\left(4\right)}$ and the principle of constant thermodynamic speed [37] to assign the times as

(30) $$\tau^{\left(j\right)}=\frac{L^{\left(j\right)}}{L_{cycle}}\tau .$$

Once $\tau^{\left(i\right)}$ is known, then the number of available steps along the leg is given by

(31) $$N^{\left(j\right)}=\frac{\tau^{\left(j\right)}}{\Delta t^{\left(j\right)}}.$$

However, if the relaxation times vary between the legs—or more generally, vary along the legs as function of κ and T, i.e., ${\left(\Delta t\right)}_{st}={\left(\Delta t\right)}_{st}\left(\kappa ,T\right)$—, we need to be more circumspect. Assuming that ${\left(\Delta t\right)}_{st}\left(\kappa ,T\right)$ is known, in a first step, we would evaluate the average relaxation time along the path, ${\left(\Delta t\right)}_{av}$, which allows us to compute an estimate of the number of discrete steps along the path $N_{st}=\tau /{\left(\Delta t\right)}_{av}$. With this information, we can use Equation (25) to compute the optimal length of each sub-piece i, ${\left(\Delta L\right)}_{i}={\left(\Delta L\right)}_{opt}=\frac{L_{cycle}}{N_{st}}=\frac{L_{cycle}}{\tau }{\left(\Delta t\right)}_{av}$. By construction, the value of ${\left(\Delta L\right)}_{opt}$ is independent of the position $\left(\kappa_{i},T_{i}\right)$ of sub-piece i along the path. Since the relaxation time varies with position i, we now need to assign each sub-piece i its own time $\tau \left(\kappa_{i},T_{i}\right)=\tau_{i}={\left(\Delta t\right)}_{st}\left(\kappa_{i},T_{i}\right)$. As long as the relaxation times do not vary much along the path, the sum of the assigned times $\sum_{i=1}^{N_{st}}\tau_{i}=\tau_{cycle}$ will nearly equal the total available time $\tau_{cycle}\approx \tau$, and the result is self-consistent. Otherwise, we need to set up a feedback loop and re-compute $N_{st}$, via, e.g., $N_{st}^{\left(k+1\right)}=N_{st}^{\left(k\right)}\frac{\tau }{\tau_{cycle}^{\left(k\right)}}\;,$ and then assign $\tau_{i}$, until self-consistency has been reached, i.e., $\sum_{i=1}^{N_{st}^{\left(k\right)}}\tau_{i}^{\left(k\right)}=\tau_{cycle}^{\left(k\right)}=\tau$. For further discussions concerning the influence of varying relaxation times in step optimal control processes, we refer to the literature [35,36].

Note that this derivation did not depend on the specific features of the hydrogen atom-like system. Thus, the minimal thermodynamic length criterion of optimality we have derived is valid for general statistical mechanical systems, whose Hamiltonian is controlled by a set of parameters $\vec{\kappa }=\left(\kappa_{a},\kappa_{b},\ldots \right)$. In this context, we note that the metric ${\overset{↔}{M}}_{Q}$ at each point i along the path is proportional to $T_{i}$, and thus we expect that the branches (j) (or parts thereof) that lie in a high temperature region, should exhibit larger thermodynamic lengths $L_{Q}^{\left(j\right)}$ than those at lower temperatures since the formulas for those lengths contain $\sqrt{T_{i}}$ as factors in each term or integral.

We now turn to the three special cases of iso-κ, isothermal, and isentropic (adiabatic and special adiabatic) paths.

### 3.2. Finite-Time Optimal Control along Isothermal Legs

For the legs with isothermal change in the parameter κ, Equation (21) yields for the excess heat $I_{Q;iso-T}\approx k_{B}T\sum_{i=1}^{N_{\kappa }}\left({\vec{\pi }}_{i}-{\vec{\pi }}_{i-1}\right)\cdot \left\{\frac{1}{{\overset{↔}{\pi }}_{i}}\right\}\left({\vec{\pi }}_{i}-{\vec{\pi }}_{i-1}\right)$ and similarly, Equation (22) for the entropy production $I_{S;iso-T}\approx k_{B}\sum_{i=1}^{N_{\kappa }}\left({\vec{\pi }}_{i}-{\vec{\pi }}_{i-1}\right)\cdot \left\{\frac{1}{{\overset{↔}{\pi }}_{i}}\right\}\left({\vec{\pi }}_{i}-{\vec{\pi }}_{i-1}\right)=I_{Q;iso-T}/T$, where $N_{\kappa }$ is the number of changes in the value of κ along the branch, which are feasible during the finite time τ assuming that each κ change takes at least a time of ${\left(\Delta t\right)}_{\kappa }$, i.e., $\tau =N_{\kappa }{\left(\Delta t\right)}_{\kappa }$. Again, $I_{Q/S}$ is minimized by picking the stopping points along the path such that the thermodynamic lengths

(32) $${\left(\Delta L_{Q}\right)}_{i}=\sqrt{k_{B}T}{\left[\left({\vec{\pi }}_{i}-{\vec{\pi }}_{i-1}\right)\left\{\frac{1}{{\overset{↔}{\pi }}_{i}}\right\}\left({\vec{\pi }}_{i}-{\vec{\pi }}_{i-1}\right)\right]}^{1/2}\;\;\mathrm{or}\;{\left(\Delta L_{S}\right)}_{i}=\sqrt{k_{B}}{\left[\left({\vec{\pi }}_{i}-{\vec{\pi }}_{i-1}\right)\left\{\frac{1}{{\overset{↔}{\pi }}_{i}}\right\}\left({\vec{\pi }}_{i}-{\vec{\pi }}_{i-1}\right)\right]}^{1/2},$$

respectively, of all $N_{\kappa }$ pieces of the path are equal,

(33) $${\left(\Delta L_{Q/S}\right)}_{i}=\frac{{\left(\Delta L_{Q/S}\right)}_{total}}{N_{\kappa }}.$$

We realize that the change in κ can be interpreted as the change in the Hamiltonian of the system. Thus, the isothermal branch of the cycle for a system with only one control parameter κ represents a special case of the general estimates of the difference between computational work and the free energy (at constant temperature)—which is computed via various thermodynamic integration methods [39,40,41] between two different Hamiltonians in finite time—, for which the optimal control problem has been analyzed in earlier work [35]. In this earlier study, it had been found that as long as we can stay close to the equilibrium distribution such that we can assume that the actual probability distribution ${\vec{p}}_{i}$ at step i equals the equilibrium probability distribution ${\vec{\pi }}_{i-1}$ at the previous step i−1, the amount of excess work along an isothermal leg is given by

(34) $$I_{iso-T}=W_{iso-T}-\Delta F_{iso-T}=\int_{0}^{\tau }\dot{\vec{E}}\left(\vec{p}-\vec{\pi }\right)dt=k_{B}T\int_{0}^{\tau }\dot{\vec{\pi }}\cdot \left\{\frac{1}{\overset{↔}{\pi }}\right\}\cdot \left(\vec{\pi }-\vec{p}\right)dt\approx \;\sum_{i=1}^{N_{\kappa }}\left({\vec{\pi }}_{i}-{\vec{\pi }}_{i-1}\right)\cdot \left\{\frac{k_{B}T}{{\overset{↔}{\pi }}_{i}}\right\}\left({\vec{\pi }}_{i}-{\vec{p}}_{i}\right)\approx k_{B}T\sum_{i=1}^{N_{\kappa }}\left({\vec{\pi }}_{i}-{\vec{\pi }}_{i-1}\right)\cdot \left\{\frac{1}{{\overset{↔}{\pi }}_{i}}\right\}\left({\vec{\pi }}_{i}-{\vec{\pi }}_{i-1}\right).$$

However, this is just the same expression as $I_{Q;iso-T}$, which we have obtained for the excess heat along an isothermal leg in (κ,T)-space, as one would have already expected from energy conservation. Since the temperature is constant along the path, we can pull T as a factor in front of the summation when computing the excess heat, and thus the thermodynamic length $L_{Q}$ of an isothermal leg (j) is proportional to $\sqrt{T^{\left(j\right)}}$. Nevertheless, since the matrix $\left\{\frac{1}{{\overset{↔}{\pi }}_{i}}\right\}$ varies along the path, the computation of the thermodynamic length is not trivial in general. 

Equations (28) and (29) can be specialized to an isothermal path by replacing λ by κ, and explicitly computing

(35) $$\frac{\partial \pi_{k}}{\partial \kappa }=-\frac{1}{k_{B}T}\pi_{k}\left[\frac{\partial E_{k}}{\partial \kappa }-\sum_{j}^{}\frac{\partial E_{j}}{\partial \kappa }\pi_{j}\right]=-\frac{1}{k_{B}T}\pi_{k}\left[\frac{\partial E_{k}}{\partial \kappa }-\vec{\left(\frac{\partial E}{\partial \kappa }\right)}\cdot \vec{\pi }\right]$$

and

(36) $$\sum_{k=1}^{N_{S}}\frac{1}{{\left(\pi \left(\lambda \right)\right)}_{k}}{\left(\frac{{\left(d\pi \left(\lambda \right)\right)}_{k}}{d\lambda }\right)}^{2}=\sum_{k=1}^{N_{S}}\frac{1}{\pi_{k}}{\left(-\frac{1}{k_{B}T}\pi_{k}\left[\frac{\partial E_{k}}{\partial \kappa }-\vec{\left(\frac{\partial E}{\partial \kappa }\right)}\cdot \vec{\pi }\right]\right)}^{2}=\frac{1}{{\left(k_{B}T\right)}^{2}}\sum_{k=1}^{N_{S}}\left(\pi_{k}{\left[\frac{\partial E_{k}}{\partial \kappa }-\;\vec{\left(\frac{\partial E_{j}}{\partial \kappa }\right)}\cdot \vec{\pi }\right]}^{2}\right)=\frac{1}{{\left(k_{B}T\right)}^{2}}\left(\vec{\pi }\cdot \vec{{\left(\frac{\partial E}{\partial \kappa }\right)}^{2}}-{\left(\vec{\left(\frac{\partial E}{\partial \kappa }\right)}\cdot \vec{\pi }\right)}^{2}\right)=\frac{1}{{\left(k_{B}T\right)}^{2}}Var_{\vec{\pi }}\left(\frac{\partial E}{\partial \kappa }\right).$$

Plugging this into Equations (28) and (29), we obtain

(37) $$L_{Q;iso-T}^{\left(j\right)}=\left|\frac{1}{\sqrt{k_{B}T}}\int_{\kappa^{\left[j\right]}}^{\kappa^{\left[j+1\right]}}\sqrt{Var_{\vec{\pi }}\left(\frac{\partial E}{\partial \kappa }\right)}d\kappa \right|\;\mathrm{and}\;L_{S;iso-T}^{\left(j\right)}=\left|\frac{1}{\sqrt{k_{B}T^{2}}}\int_{\kappa^{\left[j\right]}}^{\kappa^{\left[j+1\right]}}\sqrt{Var_{\vec{\pi }}\left(\frac{\partial E}{\partial \kappa }\right)}d\kappa \right|,$$

respectively. Note that the term under the square root is the variance of $\frac{\partial E}{\partial \kappa }$ with respect to the equilibrium probability distribution $\vec{\pi }\left(\kappa ,T\right)$, $Var_{\vec{\pi }}\left(\frac{\partial E}{\partial \kappa }\right)$, as function of T and κ.

### 3.3. Finite-Time Optimal Control along Iso-κ Legs

Next, we turn to the case of changing temperature while keeping κ constant. Along those branches, only the temperature varies, but we cannot directly profit from the fact that κ is constant when computing the excess heat. Equations (21) and (22) yield $I_{Q;iso-\kappa }\approx \sum_{i=1}^{N_{T}}\left({\vec{\pi }}_{i}-{\vec{\pi }}_{i-1}\right)\cdot \left\{\frac{k_{B}T_{i}}{{\overset{↔}{\pi }}_{i}}\right\}\left({\vec{\pi }}_{i}-{\vec{\pi }}_{i-1}\right)$ and $I_{S;iso-\kappa }\approx \sum_{i=1}^{N_{T}}\left({\vec{\pi }}_{i}-{\vec{\pi }}_{i-1}\right)\cdot \left\{\frac{k_{B}}{{\overset{↔}{\pi }}_{i}}\right\}\left({\vec{\pi }}_{i}-{\vec{\pi }}_{i-1}\right)$, respectively, where $N_{T}$ is the number of changes in the value of the temperature T along the branch, which are feasible during the finite time τ, assuming that each temperature change takes at least a time of ${\left(\Delta t\right)}_{T}$, i.e., $\tau =N_{T}{\left(\Delta t\right)}_{T}$. 

Again, we see that finding the optimal solution of distributing $N_{T}$ temperature changes over the path corresponds to minimizing $I_{Q/S}$, where the thermodynamic length of each piece i of the path is

(38) $${\left(\Delta L_{Q}\right)}_{i}={\left[\left({\vec{\pi }}_{i}-{\vec{\pi }}_{i-1}\right)\left\{\frac{k_{B}T_{i}}{{\overset{↔}{\pi }}_{i}}\right\}\left({\vec{\pi }}_{i}-{\vec{\pi }}_{i-1}\right)\right]}^{1/2}\;\mathrm{or}\;{\left(\Delta L_{S}\right)}_{i}={\left[\left({\vec{\pi }}_{i}-{\vec{\pi }}_{i-1}\right)\left\{\frac{k_{B}}{{\overset{↔}{\pi }}_{i}}\right\}\left({\vec{\pi }}_{i}-{\vec{\pi }}_{i-1}\right)\right]}^{1/2},$$

respectively. To do so, we first minimize the thermodynamic length of the complete path, ${\left(\Delta L_{Q/S}\right)}_{total}=\sum_{i=1}^{N_{T}}{\left(\Delta L_{Q/S}\right)}_{i}$, and then pick the size of each step such that they all have the same thermodynamic length

(39) $${\left(\Delta L_{Q/S}\right)}_{i}=\frac{{\left(\Delta L_{Q/S}\right)}_{total}}{N_{T}}.$$

Equations (28) and (29) can be specialized to an iso-κ path by replacing λ by T, switching variables to $\beta =\frac{1}{k_{B}T}$ and explicitly computing

(40) $$\frac{\partial \pi_{k}}{\partial T}=\frac{\partial \beta }{\partial T}\frac{\partial \pi_{k}}{\partial \beta }=-\frac{1}{k_{B}T^{2}}\left(-\pi_{k}\left[E_{k}-\sum_{j}^{}E_{j}\pi_{j}\right]\right)=\frac{1}{k_{B}T^{2}}\pi_{k}\left[E_{k}-\vec{E}\cdot \vec{\pi }\right]$$

and

(41) $$\sum_{k=1}^{N_{S}}\frac{1}{{\left(\pi \left(\lambda \right)\right)}_{k}}{\left(\frac{{\left(d\pi \left(\lambda \right)\right)}_{k}}{d\lambda }\right)}^{2}=\frac{1}{{\left(k_{B}T^{2}\right)}^{2}}\left(\vec{\pi }\cdot \vec{{\left(E\right)}^{2}}-{\left(\vec{E}\cdot \vec{\pi }\right)}^{2}\right)=\frac{1}{{\left(k_{B}T^{2}\right)}^{2}}Var_{\vec{\pi }}\left(E\right),$$

where $Var_{\vec{\pi }}\left(E\right)$ is the variance in energy E(κ) with respect to the probability distribution $\vec{\pi }$. Plugging this into Equation (28), we obtain

(42) $$L_{Q;iso-\kappa }^{\left(j\right)}=\left|\int_{T^{\left[j\right]}}^{T^{\left[j+1\right]}}\frac{\sqrt{k_{B}T}}{\sqrt{k_{B}T^{2}}}\sqrt{\frac{Var_{\vec{\pi }}\left(E\right)}{k_{B}T^{2}}}dT\right|=\left|\int_{T^{\left[j\right]}}^{T^{\left[j+1\right]}}\sqrt{\frac{C\left(\kappa ,T\right)}{T}}dT\right|\;\;\mathrm{or}\;L_{S;iso-\kappa }^{\left(j\right)}=\left|\int_{T^{\left[j\right]}}^{T^{\left[j+1\right]}}\sqrt{\frac{C\left(\kappa ,T\right)}{T^{2}}}dT\right|,$$

respectively, where $C\left(\kappa ,T\right)=\frac{1}{k_{B}T^{2}}Var_{\vec{\pi }}\left(E\right)$ is the specific heat at T and κ. Note that these expressions are reminiscent of optimal control results for the minimal entropy/excess heat production for paths where we change the temperature of a thermodynamic system in finite time [36,42]. Furthermore, for iso-κ legs along which the specific heat is approximately constant, we find $L_{Q;iso-\kappa }^{\left(j\right)}\sim \left(\sqrt{T^{\left[j+1\right]}}-\sqrt{T^{\left[j\right]}}\right)$.

### 3.4. Finite-Time Optimal Control for Adiabatic Legs

#### 3.4.1. General Adiabatic Paths

Clearly, minimizing the excess heat along a general isentropic adiabatic path is just another special case of the finite-time optimal control along general paths in (κ,T)-space. Thus, we find for the excess heat the expression $I_{Q;iso-S}\approx \sum_{i=1}^{N_{a}}\left({\vec{\pi }}_{i}-{\vec{\pi }}_{i-1}\right)\cdot \left\{\frac{k_{B}T_{i}}{{\overset{↔}{\pi }}_{i}}\right\}\left({\vec{\pi }}_{i}-{\vec{\pi }}_{i-1}\right)$ and for the entropy production $I_{S;iso-S}\approx \sum_{i=1}^{N_{a}}\left({\vec{\pi }}_{i}-{\vec{\pi }}_{i-1}\right)\cdot \left\{\frac{k_{B}}{{\overset{↔}{\pi }}_{i}}\right\}\left({\vec{\pi }}_{i}-{\vec{\pi }}_{i-1}\right)$, where $N_{a}$ is the number of changes in the values of (κ,T(κ)), or equivalently (κ(T),T) along the branch, which are feasible during the finite time τ assuming that each change in (κ,T) requires at least a time of ${\left(\Delta t\right)}_{a}$, i.e., $\tau =N_{a}{\left(\Delta t\right)}_{a}$. We keep in mind that any change in κ automatically determines a corresponding change in T such that the thermodynamic equilibrium entropy after the move has not changed, or conversely, changing the temperature enforces an appropriate change in κ. 

Again, $I_{Q/S}$ is minimized by picking the stopping points along the path such that the thermodynamic lengths

(43) $${\left(\Delta L_{Q}\right)}_{i}={\left[\left({\vec{\pi }}_{i}-{\vec{\pi }}_{i-1}\right)\left\{\frac{k_{B}T_{i}}{{\overset{↔}{\pi }}_{i}}\right\}\left({\vec{\pi }}_{i}-{\vec{\pi }}_{i-1}\right)\right]}^{1/2}\;\mathrm{or}\;{\left(\Delta L_{S}\right)}_{i}={\left[\left({\vec{\pi }}_{i}-{\vec{\pi }}_{i-1}\right)\left\{\frac{k_{B}}{{\overset{↔}{\pi }}_{i}}\right\}\left({\vec{\pi }}_{i}-{\vec{\pi }}_{i-1}\right)\right]}^{1/2},$$

respectively, of all $N_{a}$ pieces of the path are equal:

(44) $${\left(\Delta L_{Q/S}\right)}_{i}=\frac{{\left(\Delta L_{Q/S}\right)}_{total}}{N_{a}}.$$

However, for a general adiabatic path, no system independent simplification of Equations (28) and (29) is possible, since both κ and T change along such a path and their relationship T(κ) varies from system to system.

#### 3.4.2. Special Adiabatic Paths

As mentioned above, there are physical systems where not only the equilibrium entropy but also the equilibrium probability distribution of the occupancy of the microstates does not change along an adiabatic isentropic path. For such special adiabatic paths, adiabaticity is equivalent to a constant equilibrium probability distribution along the path, i.e., ${\vec{\pi }}_{i}$ = constant. On the other hand, our assumption that ${\vec{p}}_{i}$ is close to ${\vec{\pi }}_{i}$ implied that ${\vec{p}}_{i}$ can be approximated by ${\vec{p}}_{i}\approx {\vec{\pi }}_{i-1}$, and thus, to first order in $\left(\vec{\pi }\left(t\right)-\vec{p}\left(t\right)\right)$, we find for a special adiabatic path,

(45) $$I_{Q;iso-S}=Q_{iso-S;\vec{\pi }}-Q_{iso-S;\vec{p}}=0,$$

i.e., along such a leg there is no excess heat being produced due to finite time effects. We can see this also directly from the general expression for the thermodynamic length of the general adiabatic path:

(46) $${\left(\Delta L_{Q}\right)}_{total}=\sum_{i=1}^{N_{a}}{\left(\Delta L\right)}_{i}=\sum_{i=1}^{N_{a}}\sqrt{\left({\vec{\pi }}_{i}-{\vec{\pi }}_{i-1}\right)\cdot \left\{\frac{k_{B}T_{i}}{{\overset{↔}{\pi }}_{i}}\right\}\left({\vec{\pi }}_{i}-{\vec{\pi }}_{i-1}\right)}=\;\sum_{i=1}^{N_{a}}\sqrt{\left({\vec{\pi }}_{i}-{\vec{\pi }}_{i}\right)\cdot \left\{\frac{k_{B}T_{i}}{{\overset{↔}{\pi }}_{i}}\right\}\left({\vec{\pi }}_{i}-{\vec{\pi }}_{i}\right)}=0,$$

since ${\vec{\pi }}_{i}={\vec{\pi }}_{i-1}$ along a special adiabatic path, and analogously ${\left(\Delta L_{S}\right)}_{total}=0$. Here, $N_{a}$ is the number of steps along the special adiabatic path. As a consequence, the work performed by the system on the apparatus equals the negative of the energy difference between the two end points of leg (j), $W_{j\to j+1}=-{\left(\Delta E\right)}_{j\to j+1}=-\left({\langle E\rangle }^{\left[j+1\right]}-{\langle E\rangle }^{\left[j\right]}\right)$, regardless of whether we were restricted to finite time or not, and thus no excess work or heat is present, at least to first order under the assumptions we have made. Even when including higher orders, the fact that ${\vec{\pi }}_{i}$ is constant along the whole path together with ${\vec{p}}_{i=0}={\vec{\pi }}_{i=0}$ ensures that all higher order terms vanish because no relaxation of ${\vec{p}}_{i}$ to ${\vec{\pi }}_{i}$ is ever needed, thus also eliminating the issue of incomplete relaxation to equilibrium discussed in refs. [35,36].

The way finite-time considerations can enter the estimates along a special adiabatic branch must therefore involve deviations of the control itself from the true adiabatic path. Note that we did not consider such deviations from the target curve of the control parameters for the isothermal or the iso-κ branches. Since, in general, any motion of the system along these two types of branches already involves substantial changes in the equilibrium probability distribution, ${\vec{\pi }}_{i}\neq {\vec{\pi }}_{i-1}$, one usually assumes that the additional effects related to being “off-target” can be ignored in the analysis, although the issue of keeping the control parameters “on target” might be quite relevant in practical applications!

However, since ${\vec{\pi }}_{i}\equiv {\vec{\pi }}_{i-1}$ along the ideal special adiabatic path, deviations in the control parameters now become the primary source of entropy or excess heat production; other possible sources such as equilibrium fluctuations in the probability distribution ${\vec{p}}_{i}$ about ${\vec{\pi }}_{i}$ will not be considered here. As we have no knowledge about the way the control would be established in practice (i.e., in the experiment), we make the reasonable assumption that trying to stay as close as possible to the ideal adiabatic values of the control requires us again to maximize the number of change steps. This is analogous to how we proceeded earlier, where we assumed that the optimal solution of minimizing excess heat or entropy production for the general adiabatic, the isothermal, and the iso-κ branches would be found using the maximal number of steps feasible between the two endpoints of the leg: if the optimal solution requires fewer than the maximally possible number of steps, then we can just “throw away” the superfluous steps by assigning zero time to them and/or placing them at one of the endpoints of the branch.

**Figure 4:**
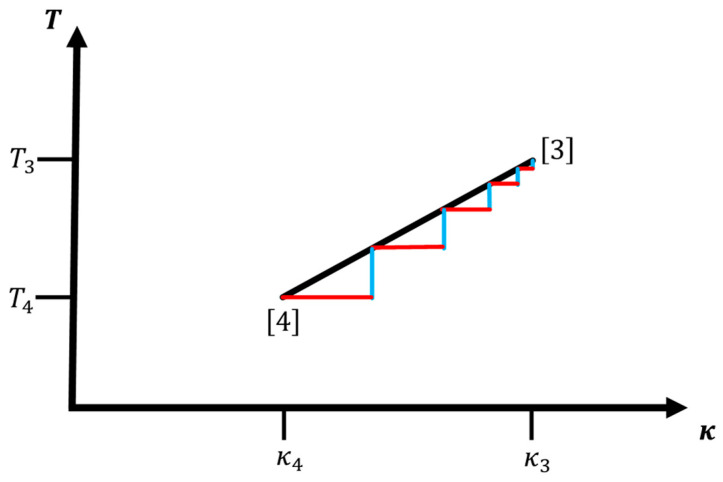
Sketch of an optimal setting of step points along a (special) adiabatic leg moving from corner [3] to corner [4], where the excess heat associated with being “off-target” with respect to the ideal adiabatic path is minimized. Note that the density of step points $\left(\kappa_{i},T_{i}\right)$ along the leg increases with temperature, approximately in a square-root fashion. Red lines are the isothermal and blue lines the iso-κ sub-pieces, respectively, which connect two points $\left(\kappa_{i-1},T_{i-1}\right)$ and $\left(\kappa_{i},T_{i}\right)$ along the perfect adiabatic path via the virtual intermediary off-target points $\left(\kappa_{i-1},T_{i}\right)$.

The maximal-number-of-steps assumption implies that we cannot perform an optimal control on the sub-step level, i.e., the deviation associated with the movement from one point i−1 with values $\left(\kappa_{i-1},T_{i-1}\right)$ along the ideal adiabatic curve to the next point i with values $\left(\kappa_{i},T_{i}\right)$ can only involve one “virtual stopping point” away from the curve “halfway” between the two curve points. In general, there exist an infinite number of choices for such “halfway points”, but from the basic modeling point of view, only two of them make sense, i.e., are consistent with our deviation analysis: $\left(\kappa_{i},T_{i-1}\right)$ “below” the adiabatic curve or $\left(\kappa_{i-1},T_{i}\right)$ “above” the adiabatic curve for paths moving from low to high temperatures; conversely, if we move from high to low temperatures, the $\left(\kappa_{i},T_{i-1}\right)$ points are “above” and the $\left(\kappa_{i-1},T_{i}\right)$ “below” the adiabatic curve (see Figure 4 below). If we could be closer to the curve than these points, then we would appear to have the ability to access temperatures and κ values in-between those of the two consecutive points along the adiabatic curve, suggesting that we have not yet maxed out the number of steps. Of course, this is only a plausibility argument and not a proof that such closer points would not be possible, but for this analysis we will employ the two virtual points given above since no further information about the actual functioning of the experimental apparatus is available. In fact, we can interpret our choice of virtual points as providing a heuristic upper bound on the entropy/excess heat production along the special adiabatic path; we essentially assume that we are able to be perfectly on target in either κ or T, and the excess production is due to the adjustment in the lagging parameter T or κ, respectively. In the following discussion, we only employ the metric ${\overset{↔}{M}}_{Q}$; completely analogous results are obtained when using the metric ${\overset{↔}{M}}_{S}$.

We note that reaching the point i via either of the virtual points requires an isothermal step and an iso-κ step. Since no sub-optimization along these steps is possible, we can directly write down the excess heat associated with each half-step and sum them. For the virtual point $\left(\kappa_{i},T_{i-1}\right)$, we have for the thermodynamic length to go from i−1 to i:

(47) $${\left(\Delta L_{Q}\right)}_{i}=\sqrt{\begin{array}{c} \left(\vec{\pi }\;\left(\kappa_{i},T_{i-1}\right)-{\vec{\pi }}_{i-1}\right)\cdot \left\{\frac{k_{B}T_{i-1}}{\overset{↔}{\pi }\left(\kappa_{i},T_{i-1}\right)}\right\}\left(\vec{\pi }\;\left(\kappa_{i},T_{i-1}\right)-{\vec{\pi }}_{i-1}\right)+\\ \left({\vec{\pi }}_{i}-\vec{\pi }\left(\kappa_{i},T_{i-1}\right)\right)\cdot \left\{\frac{k_{B}T_{i}}{{\overset{↔}{\pi }}_{i}}\right\}\left({\vec{\pi }}_{i}-\vec{\pi }\left(\kappa_{i},T_{i-1}\right)\right) \end{array}}=\;\sqrt{\begin{array}{c} \left(\vec{\pi }\;\left(\kappa_{i},T_{i-1}\right)-{\vec{\pi }}_{i}\right)\cdot \left\{\frac{k_{B}T_{i-1}}{\overset{↔}{\pi }\left(\kappa_{i},T_{i-1}\right)}\right\}\left(\vec{\pi }\;\left(\kappa_{i},T_{i-1}\right)-{\vec{\pi }}_{i}\right)+\\ \left({\vec{\pi }}_{i}-\vec{\pi }\left(\kappa_{i},T_{i-1}\right)\right)\cdot \left\{\frac{k_{B}T_{i}}{{\overset{↔}{\pi }}_{i}}\right\}\left({\vec{\pi }}_{i}-\vec{\pi }\left(\kappa_{i},T_{i-1}\right)\right) \end{array}}=\;\sqrt{\left({\vec{\pi }}_{i}-\vec{\pi }\left(\kappa_{i},T_{i-1}\right)\right)\cdot \left[\left\{\frac{k_{B}T_{i}}{{\overset{↔}{\pi }}_{i}}\right\}+\left\{\frac{k_{B}T_{i-1}}{\overset{↔}{\pi }\left(\kappa_{i},T_{i-1}\right)}\right\}\right]\left({\vec{\pi }}_{i}-\vec{\pi }\left(\kappa_{i},T_{i-1}\right)\right)},$$

where ${\vec{\pi }}_{i-1}=\vec{\pi }\;\left(\kappa_{i-1},T_{i-1}\right)$ and ${\vec{\pi }}_{i}=\vec{\pi }\;\left(\kappa_{i},T_{i}\right)$ are the two consecutive points on the adiabatic curve, and we have used the fact that ${\vec{\pi }}_{i}={\vec{\pi }}_{i-1}$. 

Similarly, for the virtual point $\left(\kappa_{i-1},T_{i}\right)$, we have

(48) $${\left(\Delta L_{Q}\right)}_{i}=\sqrt{\left({\vec{\pi }}_{i}-\vec{\pi }\left(\kappa_{i-1},T_{i}\right)\right)\cdot \left[\left\{\frac{k_{B}T_{i}}{{\overset{↔}{\pi }}_{i}}\right\}+\left\{\frac{k_{B}T_{i}}{\overset{↔}{\pi }\left(\kappa_{i-1},T_{i}\right)}\right\}\right]\left({\vec{\pi }}_{i}-\vec{\pi }\left(\kappa_{i-1},T_{i}\right)\right)}.$$

Since it is not clear which of the two virtual points would be more suitable, one might suggest that a decent approximation for ${\left(\Delta L_{Q}\right)}_{i}$ would be the average of the two thermodynamic lengths for the two points:

(49) $${\left(\Delta L_{Q}\right)}_{i}≅\frac{1}{2}(\sqrt{\left({\vec{\pi }}_{i}-\vec{\pi }\left(\kappa_{i},T_{i-1}\right)\right)\cdot \left[\left\{\frac{k_{B}T_{i}}{{\overset{↔}{\pi }}_{i}}\right\}+\left\{\frac{k_{B}T_{i-1}}{\overset{↔}{\pi }\left(\kappa_{i},T_{i-1}\right)}\right\}\right]\left({\vec{\pi }}_{i}-\vec{\pi }\left(\kappa_{i},T_{i-1}\right)\right)}+\;\sqrt{\left({\vec{\pi }}_{i}-\vec{\pi }\left(\kappa_{i-1},T_{i}\right)\right)\cdot \left[\left\{\frac{k_{B}T_{i}}{{\overset{↔}{\pi }}_{i}}\right\}+\left\{\frac{k_{B}T_{i}}{\overset{↔}{\pi }\left(\kappa_{i-1},T_{i}\right)}\right\}\right]\left({\vec{\pi }}_{i}-\vec{\pi }\left(\kappa_{i-1},T_{i}\right)\right)}).$$

However, from the point of view of optimal control, this average is not very helpful; in particular, the thermodynamic metric becomes a very complicated expression. 

Instead it is more effective to systematically pick one of the two virtual points, e.g., $\left(\kappa_{i},T_{i-1}\right)$, because this allows us to write the excess heat production due to deviations in the control from the special adiabatic path as

(50) $$I_{Q;iso-S}=\sum_{i=1}^{N_{a}}\left({\vec{\pi }}_{i}-\vec{\pi }\left(\kappa_{i},T_{i-1}\right)\right)\cdot \left[\left\{\frac{k_{B}T_{i}}{{\overset{↔}{\pi }}_{i}}\right\}+\left\{\frac{k_{B}T_{i-1}}{\overset{↔}{\pi }\left(\kappa_{i},T_{i-1}\right)}\right\}\right]\left({\vec{\pi }}_{i}-\vec{\pi }\left(\kappa_{i},T_{i-1}\right)\right)=\sum_{i=1}^{N_{a}}{\left[{\left(\Delta L_{Q}\right)}_{i}\right]}^{2},$$

where each ${\left(\Delta L_{Q}\right)}_{i}$ is the thermodynamic distance between the points i−1 and i computed with the metric $\left[\left\{\frac{k_{B}T_{i}}{{\overset{↔}{\pi }}_{i}}\right\}+\left\{\frac{k_{B}T_{i-1}}{\overset{↔}{\pi }\left(\kappa_{i},T_{i-1}\right)}\right\}\right]$, with the virtual intermediary point fixed as $\left(\kappa_{i},T_{i-1}\right)$.

We note that the probability distribution $\vec{\pi }\left(\kappa_{i},T_{i-1}\right)$ at the virtual point, which enters the expression for ${\left(\Delta L_{Q}\right)}_{i}$, is completely determined from our information about the temperatures and κ-values of the two consecutive points along the ideal special adiabatic path which are “connected” via the virtual point. Thus, there is no internal degree of freedom left for the piece of path between the points i−1 and i, which could be adjusted as part of the optimal control analysis. As a consequence, the choice of the locations of the consecutive points along the special adiabatic path completely determines ${\left(\Delta L_{Q}\right)}_{i}$, although in a slightly more involved fashion than for the isothermal and the iso-κ legs. This allows us to conclude that choosing the step points $\left(\kappa_{i},T_{i}\right)$ such that the lengths of all the ${\left(\Delta L_{Q}\right)}_{i}$ have the same value, again yields the optimal solution for minimizing the excess heat. 

While the general formula for the metric and thus the thermodynamic length of the path is more complicated than for the isothermal or iso-κ legs, we note that the metric is still essentially proportional to $k_{B}T_{i}$ at step i. Furthermore, due to the leg being special adiabatic, the term $\left[\left\{\frac{k_{B}T_{i}}{{\overset{↔}{\pi }}_{i}}\right\}+\left\{\frac{k_{B}T_{i-1}}{\overset{↔}{\pi }\left(\kappa_{i},T_{i-1}\right)}\right\}\right]$ does only weakly vary as function of the probability distribution ${\vec{\pi }}_{i}$ since the distribution is constant on the path, and should only differ slightly from ${\vec{\pi }}_{i}$ along the virtual points. Thus, we can conclude that the thermodynamic length of the virtual-point pieces between two points along the special adiabatic path mostly depends on the temperature, with

(51) $${\left(\Delta L_{Q}\right)}_{i}\sim \sqrt{T_{i}}.$$

As a consequence, the optimal distribution of points will be related to the square root of the temperature in the sense that the distance of step points along the leg (as function of temperature) will monotonically decrease as 1/$\sqrt{T_{i}}$, leading to a higher density of step points at higher temperatures (c.f. Figure 4).

## 4. Application to the Hydrogen Atom-Like System

Regarding the hydrogen atom-like system, we first note that κ and T enter the formulas for the equilibrium probability distribution everywhere in the combination

(52) $$A=\frac{\kappa }{T},$$

such that

(53) $$\pi_{k}\left(\kappa ,T\right)=\pi_{k}\left(\frac{\kappa }{T}\right)=e^{-\left(\frac{\kappa }{T}\right)\frac{E_{k}\left(1\right)}{k_{B}}}/\sum_{j=1}^{N_{S}}e^{-\left(\frac{\kappa }{T}\right)\frac{E_{j}\left(1\right)}{k_{B}}}=e^{-A\frac{E_{k}\left(1\right)}{k_{B}}}/\sum_{j=1}^{N_{S}}e^{-A\frac{E_{j}\left(1\right)}{k_{B}}}.$$

In particular, this means that the ratio $\frac{\kappa }{T}$ remains constant along the adiabatic legs of the cycle, ensuring that $\vec{\pi }$ does not change along this branch. Thus, the adiabatic branches for cycles involving the hydrogen atom-like system belong into the special category where not only the entropy but the whole equilibrium probability distribution is constant along the path. Note that in the cycle diagram (Figure 2 and Figure 3), an adiabatic branch (j) for the hydrogen atom-like system is always a straight line (that can be continued through the origin) with slope $\frac{1}{A^{\left(j\right)}}$. Of course, the actual value of the ratio A will be different for each adiabatic leg. For an adiabatic leg (j), it follows that $\kappa^{\left[j+1\right]}T^{\left[j\right]}=\kappa^{\left[j\right]}T^{\left[j+1\right]}$. For the combination of iso-κ and adiabatic legs, we have $\kappa^{\left[1\right]}=\kappa^{\left[2\right]}=\kappa_{in}$ and $\kappa^{\left[3\right]}=\kappa^{\left[4\right]}=\kappa_{f}$; therefore, we get

(54) $$\frac{\kappa_{in}}{\kappa_{f}}=\frac{T^{\left[1\right]}}{T^{\left[4\right]}}=\frac{T^{\left[2\right]}}{T^{\left[3\right]}}.$$

Analogously, for the combination of adiabatic and isothermal legs, we have the two adiabatic branches (legs 1 and 3) with $A_{1\to 2}\neq A_{3\to 4}$, for which $\kappa^{\left[1\right]}T^{\left[2\right]}=\kappa^{\left[2\right]}T^{\left[1\right]}$ and $\kappa^{\left[4\right]}T^{\left[3\right]}=\kappa^{\left[3\right]}T^{\left[4\right]}$, and the isothermal legs yield the conditions $T^{\left[3\right]}=T^{\left[2\right]}$ and $T^{\left[1\right]}=T^{\left[4\right]}$. Thus, we have the relations

(55) $$\frac{T^{\left[1\right]}}{T^{\left[2\right]}}=\frac{\kappa^{\left[1\right]}}{\kappa^{\left[2\right]}}=\frac{\kappa^{\left[4\right]}}{\kappa^{\left[3\right]}}=\frac{\kappa_{in}}{\kappa^{\left[2\right]}}=\frac{\kappa^{\left[4\right]}}{\kappa_{f}}.$$

Regarding the expressions for the thermodynamic lengths of the various legs in this section, we remark that the main effect of dealing with the hydrogen atom-like system is the special adiabaticity of the isentropic paths, such that the contribution of the adiabatic paths to the excess heat or entropy production can be set equal to zero unless we include the “off-target” contributions discussed in Section 3.4.2. In addition, we can exploit Equation (3) to rewrite the specific heat as

(56) $$C\left(\kappa ,T\right)=\frac{\vec{\pi }\cdot \vec{{\left(E\right)}^{2}}-{\left(\vec{E}\cdot \vec{\pi }\right)}^{2}}{k_{B}T^{2}}=\kappa^{2}\frac{\vec{\pi }\cdot \vec{{\left(E\left(1\right)\right)}^{2}}-{\left(\vec{E\left(1\right)}\cdot \vec{\pi }\right)}^{2}}{k_{B}T^{2}}=\kappa^{2}C^{\kappa }\left(\kappa ,T\right)=\;\frac{\kappa^{2}}{k_{B}T^{2}}Var_{\vec{\pi }}\left(E\left(1\right)\right)=\frac{A^{2}}{k_{B}}Var_{\vec{\pi }}\left(E\left(1\right)\right),$$

where both the variance $Var_{\vec{\pi }}\left(E\left(1\right)\right)=\vec{\pi }\cdot \vec{{\left(E\left(1\right)\right)}^{2}}-{\left(\vec{E\left(1\right)}\cdot \vec{\pi }\right)}^{2}=\frac{1}{\kappa^{2}}Var_{\vec{\pi }}\left(E\right)$ and the specific heat C(κ,T) are functions of $A=\left(\frac{\kappa }{T}\right)$. Furthermore, we can rewrite Equation (35) as

(57) $$\frac{\partial \pi_{k}}{\partial \kappa }=-\frac{1}{k_{B}T}\pi_{k}\left[E_{k}\left(1\right)-\sum_{j}^{}E_{j}\left(1\right)\pi_{j}\right]=-\frac{1}{k_{B}T}\pi_{k}\left[E_{k}\left(1\right)-\vec{E\left(1\right)}\cdot \vec{\pi }\right],$$

where $\pi_{k}$ is a function of $\left(\frac{\kappa }{T}\right)$, and thus Equation (36) becomes

(58) $$\frac{1}{{\left(k_{B}T\right)}^{2}}\left(\vec{\pi }\cdot \vec{E{\left(1\right)}^{2}}-{\left(\vec{E\left(1\right)}\cdot \vec{\pi }\right)}^{2}\right)=\frac{1}{k_{B}}C^{\kappa }\left(\kappa ,T\right)=\frac{1}{\kappa^{2}k_{B}}C\left(\kappa ,T\right)=\frac{Var_{\vec{\pi }}\left(E\left(1\right)\right)}{{\left(k_{B}T\right)}^{2}}.$$

Note that the variance of $\frac{\partial E}{\partial \kappa }$ in Equation (36) is now transformed into a variance of the energy E(1) of the unmodified hydrogen atom.

For each cycle discussed below, we first write down a formula for the total work done by the atom and the heat absorbed by the atom in the quasi-static approximation, where ${\vec{p}}_{i}={\vec{\pi }}_{i}$ everywhere along the cycle, and subsequently, we provide formulas for the efficiency, and the optimality criterion for the minimal excess heat production. 

### 4.1. Iso-κ-Adiabatic Cycle

In the case of the iso-κ-adiabatic cycle where the legs 2 and 4 are adiabatic and do not contribute to the heat production, we can focus on legs 1 and 3 when computing the heat exchange with the environment. Using Equations (18) and (19), we obtain therefore for the total heat exchange over the full cycle:

(59) $$Q_{cycle}=\int_{S^{\left[1\right]}}^{S^{\left[2\right]}}TdS+\int_{S^{\left[3\right]}}^{S^{\left[4\right]}}TdS=\left[T^{\left[2\right]}S^{\left[2\right]}-T^{\left[1\right]}S^{\left[1\right]}\right]+k_{B}\int_{T^{\left[1\right]}}^{T^{\left[2\right]}}\vec{\pi }\left(T,\kappa_{in}\right)\cdot ln\left(\vec{\pi }\left(T,\kappa_{in}\right)\right)dT+\;\left[T^{\left[4\right]}S^{\left[4\right]}-T^{\left[3\right]}S^{\left[3\right]}\right]+k_{B}\int_{T^{\left[3\right]}}^{T^{\left[4\right]}}\vec{\pi }\left(T,\kappa_{f}\right)\cdot ln\left(\vec{\pi }\left(T,\kappa_{f}\right)\right)dT=\vec{E}\left(\kappa_{in}\right)\cdot \left(\vec{\pi }\left(\kappa_{in}T^{\left[2\right]}\right)-\;\vec{\pi }\left(\kappa_{in}T^{\left[1\right]}\right)\right)+\vec{E}\left(\kappa_{f}\right)\cdot \left(\vec{\pi }\left(\kappa_{f}T^{\left[4\right]}\right)-\vec{\pi }\left(\kappa_{in}T^{\left[3\right]}\right)\right)=W_{cycle}.$$

Since $T^{\left[2\right]}>T^{\left[1\right]}$, $T^{\left[4\right]}<T^{\left[3\right]}$, and furthermore, the equilibrium entropy at higher temperatures is always larger than the one at lower temperatures, the term belonging to leg 1 transfers heat into the system, while along leg 3, the system gives off heat.

Since the legs 2 and 4 represent adiabatic processes, we have, furthermore, $S^{\left[2\right]}=S^{\left[3\right]}$ and $S^{\left[1\right]}=S^{\left[4\right]}$, and also $T^{\left[3\right]}=\frac{\kappa_{f}}{\kappa_{in}}T^{\left[2\right]}$ and $T^{\left[4\right]}=\frac{\kappa_{f}}{\kappa_{in}}T^{\left[1\right]}$. Using Equation (18) and shifting variables back and forth from T to $T/\kappa_{in}$ and $T/\kappa_{f}$, this implies that

(60) $$Q_{cycle}=\int_{S^{\left[1\right]},leg1}^{S^{\left[2\right]}}TdS+\int_{S^{\left[3\right]},leg3}^{S^{\left[4\right]}}TdS=\left(1-\frac{\kappa_{f}}{\kappa_{in}}\right)\left[T^{\left[2\right]}S^{\left[2\right]}-T^{\left[1\right]}S^{\left[1\right]}\right]+k_{B}\kappa_{in}\int_{T^{\left[1\right]}/\kappa_{in}}^{T^{\left[2\right]}/\kappa_{in}}\vec{\pi }\left(y\right)\cdot \;ln\left(\vec{\pi }\left(y\right)\right)dy+k_{B}\kappa_{f}\int_{T^{\left[3\right]}/\kappa_{f}}^{T^{\left[4\right]}/\kappa_{f}}\vec{\pi }\left(y\right)\cdot ln\left(\vec{\pi }\left(y\right)\right)dy=\left(1-\frac{\kappa_{f}}{\kappa_{in}}\right)\left[T^{\left[2\right]}S^{\left[2\right]}-T^{\left[1\right]}S^{\left[1\right]}\right]-\;\left(\kappa_{in}-\kappa_{f}\right)\frac{1}{\kappa_{in}}\int_{T^{\left[1\right]}}^{T^{\left[2\right]}}S\left(T\right)dT=\left(1-\frac{\kappa_{f}}{\kappa_{in}}\right)\int_{S^{\left[1\right]},leg1}^{S^{\left[2\right]}}TdS<0,$$

since $\kappa_{f}>\kappa_{in}$. This shows that the hydrogen atom-like system gives off heat to the environment, when the cycle is run as described.

Of course, this equals the total work around the cycle, which needs to balance the total heat by energy conservation. If we want to compute the expressions for the work along each of the four legs—e.g., in order to talk about the efficiency of the hydrogen atom-like system as working fluid in an engine, we need to add the contribution for the energy change. We then find: $W_{1\to 2}=W_{3\to 4}=0$, $W_{2\to 3}=-\vec{E}\left(\kappa_{f}\right)\cdot \vec{\pi }\left(\kappa_{f},T^{\left[3\right]}\right)+\vec{E}\left(\kappa_{in}\right)\cdot \vec{\pi }\left(\kappa_{in},T^{\left[2\right]}\right)=\left(\vec{E}\left(\kappa_{in}\right)-\vec{E}\left(\kappa_{f}\right)\right)\cdot \vec{\pi }\left(A_{2\to 3}\right)$, and $W_{4\to 1}=-\vec{E}\left(\kappa_{in}\right)\cdot \vec{\pi }\left(\kappa_{in},T^{\left[1\right]}\right)+\vec{E}\left(\kappa_{f}\right)\cdot \vec{\pi }\left(\kappa_{f},T^{\left[4\right]}\right)=\left(\vec{E}\left(\kappa_{f}\right)-\vec{E}\left(\kappa_{in}\right)\right)\cdot \vec{\pi }\left(A_{4\to 1}\right)$. This shows that we reduce the internal energy of the hydrogen atom-like system by a certain amount along leg 2 and raise it along leg 4 but by a much larger amount. Taking the sum, we obtain

(61) $$W_{4\to 1}+W_{2\to 3}=\left(\vec{E}\left(\kappa_{f}\right)-\vec{E}\left(\kappa_{in}\right)\right)\cdot \vec{\pi }\left(A_{4\to 1}\right)+\left(\vec{E}\left(\kappa_{in}\right)-\vec{E}\left(\kappa_{f}\right)\right)\cdot \vec{\pi }\left(A_{2\to 3}\right)\;=\left(\vec{E}\left(\kappa_{f}\right)-\vec{E}\left(\kappa_{in}\right)\right)\cdot \left(\vec{\pi }\left(A_{4\to 1}\right)-\vec{\pi }\left(A_{2\to 3}\right)\right)=\;\left(\kappa_{f}-\kappa_{in}\right)\vec{E}\left(\kappa =1\right)\cdot \left(\vec{\pi }\left(A_{4\to 1}\right)-\vec{\pi }\left(A_{2\to 3}\right)\right)<0,$$

since $\kappa_{f}>\kappa_{in}$ and $\vec{E}\left(\kappa =1\right)\cdot \vec{\pi }\left(A_{4\to 1}\right)<\vec{E}\left(\kappa =1\right)\cdot \vec{\pi }\left(A_{2\to 3}\right)<0$ because $A_{4\to 1}=\frac{\kappa_{in}}{T^{\left[1\right]}}>A_{2\to 3}=\frac{\kappa_{in}}{T^{\left[2\right]}}$. Thus, during this cycle, the apparatus performs net work on the system over the legs 2 and 4. 

For the efficiency, a possible definition is to take the ratio between the net work along the legs where κ is varied, and total heat added to/extracted from the system. With this definition, we find

(62) $$\eta_{\left\{\kappa ;S\right\}}=\frac{W_{4\to 1}+W_{2\to 3}}{Q_{cycle}}=\frac{W_{cycle}}{Q_{cycle}}=1.$$

Of course, one can define many other efficiencies, depending on the quantities and processes of interest.

Regarding the excess heat, we note that due to Equation (52), the adiabatic legs are special. We do not include the effect of being “off-target” during the adiabatic legs, and thus we only consider the two terms from the iso-κ legs 1 and 3:

(63) $$I_{Q;total}=I_{Q;1\to 2}+I_{Q;3\to 4}={\left\{\sum_{i=1}^{N_{T}}\left({\vec{\pi }}_{i}-{\vec{\pi }}_{i-1}\right)\cdot \left\{\frac{k_{B}T_{i}}{{\overset{↔}{\pi }}_{i}}\right\}\left({\vec{\pi }}_{i}-{\vec{\pi }}_{i-1}\right)\right\}}_{leg1}+{\left\{\sum_{i=1}^{N_{T}}\left({\vec{\pi }}_{i}-{\vec{\pi }}_{i-1}\right)\cdot \;\left\{\frac{k_{B}T_{i}}{{\overset{↔}{\pi }}_{i}}\right\}\left({\vec{\pi }}_{i}-{\vec{\pi }}_{i-1}\right)\right\}}_{leg3}.$$

Using Equations (42) and (56), we get for the whole cycle

(64) $$L_{Q;cycle}=L_{Q}^{\left(1\right)}+L_{Q}^{\left(3\right)}=\left|\kappa_{in}\int_{T^{\left[1\right]}}^{T^{\left[2\right]}}\sqrt{\frac{C^{\kappa }\left(\frac{\kappa }{T}\right)}{T}}dT\right|+\left|\kappa_{f}\int_{T^{\left[3\right]}}^{T^{\left[4\right]}}\sqrt{\frac{C^{\kappa }\left(\frac{\kappa }{T}\right)}{T}}dT\right|=\;|\kappa_{in}\int_{T^{\left[1\right]}}^{T^{\left[2\right]}}\sqrt{\frac{Var_{\vec{\pi }}\left(E\left(1\right)\right)}{k_{B}T^{3}}}dT|+\left|\kappa_{f}\int_{T^{\left[3\right]}}^{T^{\left[4\right]}}\sqrt{\frac{Var_{\vec{\pi }}\left(E\left(1\right)\right)}{k_{B}T^{3}}}dT\right|.$$

Switching variables to $A=\frac{\kappa }{T}$ for the integration, keeping in mind that $Var_{\vec{\pi }}\left(E\left(1\right)\right)$ is a function of $A=\frac{\kappa }{T}$, and using $A^{\left[2\right]}=A^{\left[3\right]}$ and $A^{\left[4\right]}=A^{\left[1\right]}$ since legs (4) and (2) are adiabatic, we find

(65) $$L_{Q}^{\left(1\right)}=\left|\sqrt{\kappa_{in}}\int_{A^{\left[1\right]}}^{A^{\left[2\right]}}\sqrt{\frac{Var_{\vec{\pi }}\left(E\left(1\right)\right)}{\sqrt{k_{B}A}}}dA\right|<L_{Q}^{\left(3\right)}=\left|\sqrt{\kappa_{f}}\int_{A^{\left[1\right]}}^{A^{\left[2\right]}}\sqrt{\frac{Var_{\vec{\pi }}\left(E\left(1\right)\right)}{\sqrt{k_{B}A}}}dA\right|,$$

since $\kappa_{f}>\kappa_{in}$.

In the case of the adiabatic legs considered here, we do have the problem that, to first order, the thermodynamic length vanishes as long as we are perfectly on target with the thermodynamic controls. Thus, the formula for $\tau^{\left(j\right)}$ yields zero time for the adiabatic legs of the path, which essentially means we could jump directly from the end of the first leg (= beginning of the second leg) to the beginning of the third leg (= end of the second leg), and analogously for the fourth branch. In practice, this does not make too much sense, and we would want to employ the off-target formula derived above, even though this requires additional information from experiment, or make assumptions about the location of the virtual intermediary points discussed above. Alternatively, we can make heuristic assumptions about the (hopefully short, i.e., $\tau_{a}\ll \tau$) minimal time $\tau_{a}=\tau_{Q}^{\left(2\right)}=\tau_{Q}^{\left(4\right)}$ needed to move along the adiabatic leg without noticeable deviations from the ideal adiabatic curve in (κ,T) space and subtract these times from the total available time τ, $\tau_{iso-\kappa }=\tau -2\tau_{a}$, before assigning $\tau_{Q}^{\left(1\right)}$ and $\tau_{Q}^{\left(3\right)}$ according the principle of constant thermodynamic speed.

Since $\frac{L_{Q}^{\left(3\right)}}{L_{Q}^{\left(1\right)}}=\frac{\sqrt{\kappa_{f}}}{\sqrt{\kappa_{in}}}$, the optimal time allocation for the two legs must be

(66) $$\frac{\tau_{Q}^{\left(3\right)}}{\tau_{Q}^{\left(1\right)}}=\frac{L_{Q}^{\left(3\right)}}{L_{Q}^{\left(1\right)}}=\frac{\sqrt{\kappa_{f}}}{\sqrt{\kappa_{in}}},$$

and thus we obtain $\tau_{Q}^{\left(1\right)}=\tau_{iso-\kappa }\frac{\sqrt{\kappa_{in}}}{\sqrt{\kappa_{f}}+\sqrt{\kappa_{in}}}$ and $\tau_{Q}^{\left(3\right)}=\tau_{iso-\kappa }\frac{\sqrt{\kappa_{f}}}{\sqrt{\kappa_{f}}+\sqrt{\kappa_{in}}}$. We remark that when we minimize the entropy production and thus use the metric ${\overset{↔}{M}}_{S}$, we find $L_{S}^{\left(3\right)}=L_{S}^{\left(1\right)}$ and, after subtracting $\tau_{a}=\tau_{S}^{\left(2\right)}=\tau_{S}^{\left(4\right)}$ from τ, we obtain $\tau_{S}^{\left(1\right)}=\tau_{S}^{\left(3\right)}=\frac{\tau_{iso-\kappa }}{2}$. 

Due to the sum over microstates for the heat capacity with nontrivial energy eigenvalues under the square root and the nontrivial temperature dependence of $\vec{\pi }\left(T,\kappa \right)$, no further analytical simplifications appear to be possible. Thus, the allocation along the individual legs is an open question, but should depend on the variance $Var_{\vec{\pi }}\left(E\left(1\right)\right)$ as function of $\left(\frac{\kappa }{T}\right)$. 

### 4.2. Iso-κ-Isothermal Cycle

For the iso-κ-isothermal cycle ($T^{\left[3\right]}=T^{\left[2\right]}$ and $T^{\left[4\right]}=T^{\left[1\right]}$), we have a non-vanishing heat term along all legs, where the integrals over temperature along the isothermal branches vanish, of course,

(67) $$k_{B}\int_{T^{\left[2\right]}}^{T^{\left[3\right]}}\vec{\pi }\left(\kappa ,T\right)\cdot ln\left(\vec{\pi }\left(\kappa ,T\right)\right)dT=k_{B}\int_{T^{\left[4\right]}}^{T^{\left[1\right]}}\vec{\pi }\left(\kappa ,T\right)\cdot ln\left(\vec{\pi }\left(\kappa ,T\right)\right)dT=0.$$

Using the fact that along the iso-κ branches $Q^{\left(j\right)}={\left(\Delta E\right)}^{\left(j\right)}$, we get for the total heat produced on the cycle,

(68) $$Q_{cycle}=\vec{E}\left(\kappa_{in}\right)\cdot \left[\vec{\pi }\left(\kappa_{in},T^{\left[2\right]}\right)-\vec{\pi }\left(\kappa_{in},T^{\left[1\right]}\right)\right]+\left[T^{\left[2\right]}S^{\left[3\right]}\left(\kappa_{f},T^{\left[2\right]}\right)-T^{\left[2\right]}S^{\left[2\right]}\left(\kappa_{in},T^{\left[2\right]}\right)\right]+\;\vec{E}\left(\kappa_{f}\right)\cdot \left[\vec{\pi }\left(\kappa_{f},T^{\left[1\right]}\right)-\vec{\pi }\left(\kappa_{f},T^{\left[2\right]}\right)\right]+\left[T^{\left[1\right]}S^{\left[1\right]}\left(\kappa_{in},T^{\left[1\right]}\right)-T^{\left[1\right]}S^{\left[4\right]}\left(\kappa_{f},T^{\left[1\right]}\right)\right].$$

For the work along the legs, we find $W_{1\to 2}=W_{3\to 4}=0$, $W_{2\to 3}=\left[T^{\left[2\right]}S^{\left[3\right]}\left(\kappa_{f},T^{\left[2\right]}\right)-T^{\left[2\right]}S^{\left[2\right]}\left(\kappa_{in},T^{\left[2\right]}\right)\right]-\vec{E}\left(\kappa_{f}\right)\cdot \vec{\pi }\left(\kappa_{f},T^{\left[2\right]}\right)+\vec{E}\left(\kappa_{in}\right)\cdot \vec{\pi }\left(\kappa_{in},T^{\left[2\right]}\right)$ and $W_{4\to 1}=\left[T^{\left[1\right]}S^{\left[1\right]}\left(\kappa_{in},T^{\left[1\right]}\right)-T^{\left[1\right]}S^{\left[4\right]}\left(\kappa_{f},T^{\left[1\right]}\right)\right]-\vec{E}\left(\kappa_{in}\right)\cdot \vec{\pi }\left(\kappa_{in},T^{\left[1\right]}\right)+\vec{E}\left(\kappa_{f}\right)\cdot \vec{\pi }\left(\kappa_{f},T^{\left[1\right]}\right)$. The possible net extracted work from the isothermal legs 2 and 4 is now:

(69) $$W_{4\to 1}+W_{2\to 3}=\left[T^{\left[2\right]}S^{\left[3\right]}\left(\kappa_{f},T^{\left[2\right]}\right)-T^{\left[2\right]}S^{\left[2\right]}\left(\kappa_{in},T^{\left[2\right]}\right)+T^{\left[1\right]}S^{\left[1\right]}\left(\kappa_{in},T^{\left[1\right]}\right)-\;T^{\left[1\right]}S^{\left[4\right]}\left(\kappa_{f},T^{\left[1\right]}\right)\right]+\vec{E}\left(1\right)\cdot \left\{\kappa_{f}\left[\vec{\pi }\left(\kappa_{f},T^{\left[1\right]}\right)-\vec{\pi }\left(\kappa_{f},T^{\left[2\right]}\right)\right]-\kappa_{in}\left[\vec{\pi }\left(\kappa_{in},T^{\left[1\right]}\right)-\vec{\pi }\left(\kappa_{in},T^{\left[2\right]}\right)\right]\right\}.$$

Now, $\frac{\kappa_{f}}{T^{\left[2\right]}}>\frac{\kappa_{in}}{T^{\left[2\right]}}$ and similarly $\frac{\kappa_{f}}{T^{\left[1\right]}}>\frac{\kappa_{in}}{T^{\left[1\right]}}$, and thus the probability distribution $\vec{\pi }\left(\kappa_{f},T^{\left[2\right]}\right)$ is more concentrated at low energies than $\vec{\pi }\left(\kappa_{in},T^{\left[2\right]}\right)$, leading to the conclusion that $S\left(\kappa_{f},T^{\left[2\right]}\right)<S\left(\kappa_{in},T^{\left[2\right]}\right)$, and analogously $S\left(\kappa_{f},T^{\left[1\right]}\right)<S\left(\kappa_{in},T^{\left[1\right]}\right)$. Similarly, $\vec{E}\left(1\right)\cdot \vec{\pi }\left(\kappa_{f},T^{\left[1\right]}\right)<\vec{E}\left(1\right)\cdot \vec{\pi }\left(\kappa_{f},T^{\left[2\right]}\right)$ and $\vec{E}\left(1\right)\cdot \vec{\pi }\left(\kappa_{in},T^{\left[1\right]}\right)<\vec{E}\left(1\right)\cdot \vec{\pi }\left(\kappa_{in},T^{\left[2\right]}\right)$. Since $\kappa_{f}>\kappa_{in}$, the energy contribution to the work along these legs should be negative, resulting in an overall negative work, i.e., the environment performs net work on the hydrogen atom-like system.

Again, we compute the efficiency as

(70) $$\eta_{\left\{\kappa ;T\right\}}=\frac{W^{\left(2\right)}+W^{\left(4\right)}}{Q_{cycle}}=\frac{W_{cycle}}{Q_{cycle}}=1.$$

Regarding the excess heat production, all four legs contribute in this case. Thus, we have

(71) $$I_{Q;total}=I_{Q;1\to 2}+I_{Q;2\to 3}+I_{Q;3\to 4}+I_{Q;4\to 1}={\left\{\sum_{i=1}^{N_{T}}\left({\vec{\pi }}_{i}-{\vec{\pi }}_{i-1}\right)\cdot \left\{\frac{k_{B}T_{i}}{{\overset{↔}{\pi }}_{i}}\right\}\left({\vec{\pi }}_{i}-{\vec{\pi }}_{i-1}\right)\right\}}_{leg1}+\;k_{B}T_{2}{\left\{\sum_{i=1}^{N_{\kappa }}\left({\vec{\pi }}_{i}-{\vec{\pi }}_{i-1}\right)\cdot \left\{\frac{1}{{\overset{↔}{\pi }}_{i}}\right\}\left({\vec{\pi }}_{i}-{\vec{\pi }}_{i-1}\right)\right\}}_{leg2}+{\left\{\sum_{i=1}^{N_{T}}\left({\vec{\pi }}_{i}-{\vec{\pi }}_{i-1}\right)\cdot \left\{\frac{k_{B}T_{i}}{{\overset{↔}{\pi }}_{i}}\right\}\left({\vec{\pi }}_{i}-{\vec{\pi }}_{i-1}\right)\right\}}_{leg3}\;+\;k_{B}T_{1}{\left\{\sum_{i=1}^{N_{\kappa }}\left({\vec{\pi }}_{i}-{\vec{\pi }}_{i-1}\right)\cdot \left\{\frac{1}{{\overset{↔}{\pi }}_{i}}\right\}\left({\vec{\pi }}_{i}-{\vec{\pi }}_{i-1}\right)\right\}}_{leg4}.$$

Note that *N_T_* and *N_K_* of the individual legs will be determined from the ratios $\frac{\tau^{\left(j\right)}}{{\left(\Delta t\right)}_{T,\kappa }}$, where we again will assume that the relaxation times are constant. Employing Equations (37), (42) and (56)–(58), the total thermodynamic length of the cycle is then

(72) $$L_{Q;cycle}=L_{Q}^{\left(1\right)}+L_{Q}^{\left(2\right)}+L_{Q}^{\left(3\right)}+L_{Q}^{\left(4\right)}=\left|\kappa_{in}\int_{T^{\left[1\right]}}^{T^{\left[2\right]}}\sqrt{\frac{C^{\kappa }\left(\frac{\kappa_{in}}{T}\right)}{T}}dT\right|+\left|\sqrt{T^{\left[2\right]}}\int_{\kappa_{in}}^{\kappa_{f}}\sqrt{C^{\kappa }\left(\frac{\kappa }{T^{\left[2\right]}}\right)}d\kappa \right|+\;\left|\kappa_{f}\int_{T^{\left[2\right]}}^{T^{\left[1\right]}}\sqrt{\frac{C^{\kappa }\left(\frac{\kappa_{f}}{T}\right)}{T}}dT\right|+\left|\sqrt{T^{\left[1\right]}}\int_{\kappa_{f}}^{\kappa_{in}}\sqrt{C^{\kappa }\left(\frac{\kappa }{T^{\left[1\right]}}\right)}d\kappa \right|=\left|\kappa_{in}\int_{T^{\left[1\right]}}^{T^{\left[2\right]}}\sqrt{\frac{Var_{\vec{\pi }}\left(E\left(1\right)\right)}{k_{B}T^{3}}}dT\right|+\;\left|\sqrt{T^{\left[2\right]}}\int_{\kappa_{in}}^{\kappa_{f}}\sqrt{\frac{Var_{\vec{\pi }}\left(E\left(1\right)\right)}{k_{B}T^{2}}}d\kappa \right|+\left|\kappa_{f}\int_{T^{\left[1\right]}}^{T^{\left[2\right]}}\sqrt{\frac{Var_{\vec{\pi }}\left(E\left(1\right)\right)}{k_{B}T^{3}}}dT\right|+\left|\sqrt{T^{\left[1\right]}}\int_{\kappa_{in}}^{\kappa_{f}}\sqrt{\frac{Var_{\vec{\pi }}\left(E\left(1\right)\right)}{k_{B}T^{2}}}d\kappa \right|.$$

Keeping in mind that $Var_{\vec{\pi }}\left(E\left(1\right)\right)$ depends only on A, we switch variables in all integrals from T and κ to $A=\frac{\kappa }{T}$, to obtain

(73) $$L_{Q}^{\left(1\right)}+L_{Q}^{\left(2\right)}+L_{Q}^{\left(3\right)}+L_{Q}^{\left(4\right)}=\left|\sqrt{\kappa_{in}}\int_{A^{\left[1\right]}}^{A^{\left[2\right]}}\sqrt{\frac{Var_{\vec{\pi }}\left(E\left(1\right)\right)}{k_{B}A}}dA\right|+\left|\sqrt{T^{\left[2\right]}}\int_{A^{\left[2\right]}}^{A^{\left[3\right]}}\sqrt{\frac{Var_{\vec{\pi }}\left(E\left(1\right)\right)}{k_{B}}}dA\right|+\;\left|\sqrt{\kappa_{f}}\int_{A^{\left[3\right]}}^{A^{\left[4\right]}}\sqrt{\frac{Var_{\vec{\pi }}\left(E\left(1\right)\right)}{k_{B}A}}dA\right|+\left|\sqrt{T^{\left[1\right]}}\int_{A^{\left[4\right]}}^{A^{\left[1\right]}}\sqrt{\frac{Var_{\vec{\pi }}\left(E\left(1\right)\right)}{k_{B}}}dA\right|.$$

Note that since all legs contribute already to first order to the total thermodynamic length, the times spent in each leg in the optimal case are proportional to the thermodynamic lengths of each leg, $\tau_{Q}^{\left(j\right)}=\frac{L_{Q}^{\left(j\right)}}{L_{cycle}}\tau$. Since the values of A at the corners are all different, no simple general estimate of assigning times to the various legs appears possible. However, for the special case of (approximately) constant variance $Var_{\vec{\pi }}\left(E\left(1\right)\right)$, we find $L_{Q}^{\left(1\right)}=\kappa_{in}\left(\frac{1}{\sqrt{T_{1}}}-\frac{1}{\sqrt{T_{2}}}\right)\sqrt{\frac{Var_{\vec{\pi }}\left(E\left(1\right)\right)}{k_{B}}}$, $L_{Q}^{\left(2\right)}=\left(\frac{\kappa_{f}}{\sqrt{T_{2}}}-\frac{\kappa_{in}}{\sqrt{T_{2}}}\right)\sqrt{\frac{Var_{\vec{\pi }}\left(E\left(1\right)\right)}{k_{B}}}$, $L^{\left(3\right)}=\kappa_{f}\left(\frac{1}{\sqrt{T_{1}}}-\frac{1}{\sqrt{T_{2}}}\right)\sqrt{\frac{Var_{\vec{\pi }}\left(E\left(1\right)\right)}{k_{B}}}$ and $L_{Q}^{\left(4\right)}=\left(\frac{\kappa_{f}}{\sqrt{T_{1}}}-\frac{\kappa_{in}}{\sqrt{T_{1}}}\right)\sqrt{\frac{Var_{\vec{\pi }}\left(E\left(1\right)\right)}{k_{B}}}$. From this follows

(74) $$L_{Q;cycle}=2\sqrt{\frac{Var_{\vec{\pi }}\left(E\left(1\right)\right)}{k_{B}}}\left(\frac{\kappa_{f}}{\sqrt{T_{1}}}-\frac{\kappa_{in}}{\sqrt{T_{2}}}\right)=2\sqrt{\frac{Var_{\vec{\pi }}\left(E\left(1\right)\right)}{k_{B}}}\frac{\left(\sqrt{T_{2}}\kappa_{f}-\sqrt{T_{1}}\kappa_{in}\right)}{\sqrt{T_{1}T_{2}}},$$

and thus the optimal times for the four legs we obtain $\tau_{Q}^{\left(1\right)}=\frac{\tau }{2}\left[\frac{\sqrt{T_{2}}\kappa_{in}-\sqrt{T_{1}}\kappa_{in}}{\sqrt{T_{2}}\kappa_{f}-\sqrt{T_{1}}\kappa_{in}}\right]$, $\tau_{Q}^{\left(2\right)}=\frac{\tau }{2}\left[\frac{\sqrt{T_{1}}\kappa_{f}-\sqrt{T_{1}}\kappa_{in}}{\sqrt{T_{2}}\kappa_{f}-\sqrt{T_{1}}\kappa_{in}}\right]$, $\tau_{Q}^{\left(3\right)}=\frac{\tau }{2}\left[\frac{\sqrt{T_{2}}\kappa_{f}-\sqrt{T_{1}}\kappa_{f}}{\sqrt{T_{2}}\kappa_{f}-\sqrt{T_{1}}\kappa_{in}}\right]$ and $\tau_{Q}^{\left(4\right)}=\frac{\tau }{2}\left[\frac{\sqrt{T_{2}}\kappa_{f}-\sqrt{T_{2}}\kappa_{in}}{\sqrt{T_{2}}\kappa_{f}-\sqrt{T_{1}}\kappa_{in}}\right]$. Analogous results are obtained using the metric ${\overset{↔}{M}}_{S}$ for the case of minimum entropy production. In particular, if we again assume an approximately constant variance $Var_{\vec{\pi }}\left(E\left(1\right)\right)$, we obtain $L_{S}^{\left(1\right)}=\left(\frac{\kappa_{in}}{T_{1}}-\frac{\kappa_{in}}{T_{2}}\right)\sqrt{\frac{Var_{\vec{\pi }}\left(E\left(1\right)\right)}{k_{B}}}$, $L_{Q}^{\left(2\right)}=\left(\frac{\kappa_{f}}{T_{2}}-\frac{\kappa_{in}}{T_{2}}\right)\sqrt{\frac{Var_{\vec{\pi }}\left(E\left(1\right)\right)}{k_{B}}}$, $L^{\left(3\right)}=\left(\frac{\kappa_{f}}{T_{1}}-\frac{\kappa_{f}}{T_{2}}\right)\sqrt{\frac{Var_{\vec{\pi }}\left(E\left(1\right)\right)}{k_{B}}}$ and $L_{Q}^{\left(4\right)}=\left(\frac{\kappa_{f}}{T_{1}}-\frac{\kappa_{in}}{T_{1}}\right)\sqrt{\frac{Var_{\vec{\pi }}\left(E\left(1\right)\right)}{k_{B}}}$. From this follows for the total thermodynamic length of the path $L_{S;cycle}=2\sqrt{\frac{Var_{\vec{\pi }}\left(E\left(1\right)\right)}{k_{B}}}\frac{\left(T_{2}\kappa_{f}-T_{1}\kappa_{in}\right)}{T_{1}T_{2}}$, yielding the optimal time assignments to the legs as $\tau_{S}^{\left(1\right)}=\frac{\tau }{2}\left[\frac{T_{2}\kappa_{in}-T_{1}\kappa_{in}}{T_{2}\kappa_{f}-T_{1}\kappa_{in}}\right]$, $\tau_{S}^{\left(2\right)}=\frac{\tau }{2}\left[\frac{T_{1}\kappa_{f}-T_{1}\kappa_{in}}{T_{2}\kappa_{f}-T_{1}\kappa_{in}}\right]$, $\tau_{S}^{\left(3\right)}=\frac{\tau }{2}\left[\frac{T_{2}\kappa_{f}-T_{1}\kappa_{f}}{T_{2}\kappa_{f}-T_{1}\kappa_{in}}\right]$ and $\tau_{S}^{\left(4\right)}=\frac{\tau }{2}\left[\frac{T_{2}\kappa_{f}-T_{2}\kappa_{in}}{T_{2}\kappa_{f}-T_{1}\kappa_{in}}\right]$.

### 4.3. Isothermal-Adiabatic Cycle

In the case of the isothermal-adiabatic cycle where the legs 1 and 3 are adiabatic and do not contribute to the heat exchange, we have from legs 2 and 4:

(75) $$Q_{cycle}=\int_{S^{\left[2\right]}}^{S^{\left[3\right]}}TdS+\int_{S^{\left[4\right]}}^{S^{\left[1\right]}}TdS=\left[T^{\left[3\right]}S^{\left[3\right]}-T^{\left[2\right]}S^{\left[2\right]}\right]+k_{B}\int_{T^{\left[2\right]}}^{T^{\left[3\right]}}\vec{\pi }\left(T,\kappa_{in}\right)\cdot ln\left(\vec{\pi }\left(T,\kappa_{in}\right)\right)dT+\;\left[T^{\left[1\right]}S^{\left[1\right]}-T^{\left[4\right]}S^{\left[4\right]}\right]+k_{B}\int_{T^{\left[4\right]}}^{T^{\left[1\right]}}\vec{\pi }\left(T,\kappa_{f}\right)\cdot ln\left(\vec{\pi }\left(T,\kappa_{f}\right)\right)dT=T^{\left[2\right]}\left[S^{\left[3\right]}-S^{\left[1\right]}\right]+\;T^{\left[1\right]}\left[S^{\left[1\right]}-S^{\left[3\right]}\right]=\left(T^{\left[2\right]}-T^{\left[1\right]}\right)\left[S^{\left[3\right]}-S^{\left[1\right]}\right]=W_{cycle}.$$

Here, we have used the fact that the temperature does not change along legs 2 and 4, $T^{\left[2\right]}=T^{\left[3\right]}$ and $T^{\left[1\right]}=T^{\left[4\right]}$, and that the entropy does not change along the adiabatic legs, i.e., $S^{\left[1\right]}=S^{\left[2\right]}$ and $S^{\left[3\right]}=S^{\left[4\right]}$. 

Since $T^{\left[2\right]}>T^{\left[1\right]}$, and furthermore, for equal temperatures, the equilibrium entropy decreases with κ, i.e., $S^{\left[3\right]}<S^{\left[2\right]}=S^{\left[1\right]}$, we have a rejection of heat over the cycle, i.e., the hydrogen atom-like system converts work performed on it into heat transferred to the environment. Furthermore, the term belonging to leg 2 absorbs heat in the system, while along leg 3, we are removing heat from the system. 

For the work performed by the system along the four legs, we find $W_{1\to 2}=-\left(\vec{E}\left(\kappa_{2}\right)\cdot \vec{\pi }\left(\kappa_{2},T^{\left[2\right]}\right)-\vec{E}\left(\kappa_{in}\right)\vec{\pi }\left(\kappa_{in},T^{\left[1\right]}\right)\right)$, $W_{2\to 3}=T^{\left[2\right]}\left[S^{\left[3\right]}\left(\kappa_{f},T^{\left[2\right]}\right)-S^{\left[2\right]}\left(\kappa_{2},T^{\left[2\right]}\right)\right]-\left[\vec{E}\left(\kappa_{f}\right)\cdot \vec{\pi }\left(\kappa_{f},T^{\left[2\right]}\right)-\vec{E}\left(\kappa_{2}\right)\cdot \vec{\pi }\left(\kappa_{2},T^{\left[2\right]}\right)\right]$, $W_{3\to 4}=-\left(\vec{E}\left(\kappa_{4}\right)\cdot \vec{\pi }\left(\kappa_{4},T^{\left[1\right]}\right)-\vec{E}\left(\kappa_{f}\right)\vec{\pi }\left(\kappa_{f},T^{\left[2\right]}\right)\right)$, and $W_{4\to 1}=T^{\left[1\right]}\left[S^{\left[1\right]}\left(\kappa_{in},T^{\left[1\right]}\right)-S^{\left[4\right]}\left(\kappa_{4},T^{\left[1\right]}\right)\right]-\left[\vec{E}\left(\kappa_{in}\right)\cdot \vec{\pi }\left(\kappa_{in},T^{\left[1\right]}\right)-\vec{E}\left(\kappa_{4}\right)\cdot \vec{\pi }\left(\kappa_{4},T^{\left[1\right]}\right)\right]$. Taking the sum along the two isothermal legs, we obtain

(76) $$W_{4\to 1}+W_{2\to 3}=T^{\left[1\right]}\left[S^{\left[1\right]}\left(\kappa_{in},T^{\left[1\right]}\right)-S^{\left[4\right]}\left(\kappa_{4},T^{\left[1\right]}\right)\right]-\left[\vec{E}\left(\kappa_{in}\right)\cdot \vec{\pi }\left(\kappa_{in},T^{\left[1\right]}\right)-\vec{E}\left(\kappa_{4}\right)\cdot \;\vec{\pi }\left(\kappa_{4},T^{\left[1\right]}\right)\right]+T^{\left[2\right]}\left[S^{\left[3\right]}\left(\kappa_{f},T^{\left[2\right]}\right)-S^{\left[2\right]}\left(\kappa_{2},T^{\left[2\right]}\right)\right]-\left[\vec{E}\left(\kappa_{f}\right)\cdot \vec{\pi }\left(\kappa_{f},T^{\left[2\right]}\right)-\vec{E}\left(\kappa_{2}\right)\cdot \;\vec{\pi }\left(\kappa_{2},T^{\left[2\right]}\right)\right]=\left[T^{\left[2\right]}-T^{\left[1\right]}\right]\left[S^{\left[3\right]}-S^{\left[1\right]}\right]-\left(1-\frac{T_{1}}{T_{2}}\right)\kappa_{f}\vec{E}\left(\kappa =1\right)\cdot \left\{\vec{\pi }\left(A_{3\to 4}\right)-\;\frac{\kappa_{in}}{\kappa_{f}}\left(\frac{T_{2}}{T_{1}}\right)\vec{\pi }\left(A_{1\to 2}\right)\right\},$$

where we have used $S^{\left[1\right]}=S^{\left[2\right]}$, $S^{\left[3\right]}=S^{\left[4\right]}$, $A_{1\to 2}=\frac{\kappa_{in}}{T^{\left[1\right]}}=\frac{\kappa_{2}}{T^{\left[2\right]}}$, and $A_{3\to 4}=\frac{\kappa_{f}}{T^{\left[2\right]}}=\frac{\kappa_{4}}{T^{\left[1\right]}}$. We note that $\vec{E}\left(1\right)\cdot \vec{\pi }\left(A_{3\to 4}\right)<\vec{E}\left(1\right)\cdot \vec{\pi }\left(A_{1\to 2}\right)<0$ and thus

(77) $$-\kappa_{f}\left(1-\frac{T_{1}}{T_{2}}\right)\vec{E}\left(\kappa =1\right)\cdot \left\{\vec{\pi }\left(A_{3\to 4}\right)-\frac{\kappa_{in}}{\kappa_{f}}\left(\frac{T_{2}}{T_{1}}\right)\vec{\pi }\left(A_{1\to 2}\right)\right\}>0$$

as long as $\frac{\kappa_{in}}{\kappa_{f}}\left(\frac{T_{2}}{T_{1}}\right)<1$; note that this is exactly the feasibility condition for the special adiabatic-isothermal cycle, Equation (5). Furthermore, $S^{\left[3\right]}<S^{\left[1\right]}$ and thus $\left[T^{\left[2\right]}-T^{\left[1\right]}\right]\left[S^{\left[3\right]}-S^{\left[1\right]}\right]<0$, suggesting that the net work for these two legs is likely to be negative, i.e., the apparatus does work on the atom and increases its internal energy in the process. 

For the “efficiency”, we again compute the ratio between the net work along the isothermal legs and the total heat exchanged

(78) $$\eta_{\left\{S;T\right\}}^{isothermal}=\frac{W_{4\to 1}+W_{2\to 3}}{Q_{cycle}}=1-\frac{-\kappa_{f}\left(1-\frac{T_{1}}{T_{2}}\right)\vec{E}\left(\kappa =1\right)\cdot \left\{\vec{\pi }\left(A_{3\to 4}\right)-\frac{\kappa_{in}}{\kappa_{f}}\left(\frac{T_{2}}{T_{1}}\right)\vec{\pi }\left(A_{1\to 2}\right)\right\}}{\left(T^{\left[2\right]}-T^{\left[1\right]}\right)\left[S^{\left[1\right]}-S^{\left[3\right]}\right]}<1.$$

Alternatively, we could use the two adiabatic legs to extract/perform work, i.e.,

(79) $$W_{1\to 2}+W_{3\to 4}=-\left(\vec{E}\left(\kappa_{4}\right)\cdot \vec{\pi }\left(\kappa_{4},T^{\left[1\right]}\right)-\vec{E}\left(\kappa_{f}\right)\vec{\pi }\left(\kappa_{f},T^{\left[2\right]}\right)\right)-\left(\vec{E}\left(\kappa_{2}\right)\cdot \vec{\pi }\left(\kappa_{2},T^{\left[2\right]}\right)-\;\vec{E}\left(\kappa_{in}\right)\vec{\pi }\left(\kappa_{in},T^{\left[1\right]}\right)\right)=\left(1-\frac{T_{1}}{T_{2}}\right)\kappa_{f}\vec{E}\left(\kappa =1\right)\cdot \left\{\vec{\pi }\left(A_{3\to 4}\right)-\frac{\kappa_{in}}{\kappa_{f}}\left(\frac{T_{2}}{T_{1}}\right)\vec{\pi }\left(A_{1\to 2}\right)\right\};$$

in that case, we get for the efficiency

(80) $$\eta_{\left\{S;T\right\}}^{adiabatic}=\frac{W_{1\to 2}+W_{3\to 4}}{Q_{cycle}}=\frac{\left(1-\frac{T_{1}}{T_{2}}\right)\kappa_{f}\vec{E}\left(\kappa =1\right)\cdot \left\{\vec{\pi }\left(A_{3\to 4}\right)-\frac{\kappa_{in}}{\kappa_{f}}\left(\frac{T_{2}}{T_{1}}\right)\vec{\pi }\left(A_{1\to 2}\right)\right\}}{\left(T^{\left[2\right]}-T^{\left[1\right]}\right)\left[S^{\left[3\right]}-S^{\left[1\right]}\right]}.$$

Clearly, $\eta_{\left\{ad;T\right\}}^{isothermal}+\eta_{\left\{ad;T\right\}}^{adiabatic}=1$, since $W_{cycle}=Q_{cycle}$.

Regarding minimizing the excess heat, we do not include the effect of being “off-target” during the adiabatic legs, and thus we only have the two terms from the isothermal legs 2 and 4:

(81) $$I_{Q;total}=I_{Q;2\to 3}+I_{Q;4\to 1}={\left\{\sum_{i=1}^{N_{T}}\left({\vec{\pi }}_{i}-{\vec{\pi }}_{i-1}\right)\cdot \left\{\frac{k_{B}T_{i}}{{\overset{↔}{\pi }}_{i}}\right\}\left({\vec{\pi }}_{i}-{\vec{\pi }}_{i-1}\right)\right\}}_{leg2}+{\left\{\sum_{i=1}^{N_{T}}\left({\vec{\pi }}_{i}-{\vec{\pi }}_{i-1}\right)\cdot \;\left\{\frac{k_{B}T_{i}}{{\overset{↔}{\pi }}_{i}}\right\}\left({\vec{\pi }}_{i}-{\vec{\pi }}_{i-1}\right)\right\}}_{leg4}.$$

We can use Equations (37) and (58) to write an expression for the thermodynamic length of the cycle:

(82) $$L_{Q;cycle}=L_{Q}^{\left(2\right)}+L_{Q}^{\left(4\right)}=\left|\sqrt{T^{\left[2\right]}}\int_{\kappa^{\left[2\right]}}^{\kappa_{f}}\frac{1}{\kappa }\sqrt{C\left(\frac{\kappa }{T^{\left[2\right]}}\right)}d\kappa \right|+\left|\sqrt{T^{\left[1\right]}}\int_{\kappa^{\left[4\right]}}^{\kappa_{in}}\frac{1}{\kappa }\sqrt{C\left(\frac{\kappa }{T^{\left[1\right]}}\right)}d\kappa \right|=\;\left|\sqrt{T^{\left[2\right]}}\int_{\kappa^{\left[2\right]}}^{\kappa_{f}}\sqrt{\frac{Var_{\vec{\pi }}\left(E\left(1\right)\right)}{k_{B}T^{2}}}d\kappa \right|+\left|\sqrt{T^{\left[1\right]}}\int_{\kappa^{\left[4\right]}}^{\kappa_{in}}\sqrt{\frac{Var_{\vec{\pi }}\left(E\left(1\right)\right)}{k_{B}T^{2}}}d\kappa \right|.$$

We note that for the hydrogen atom-like system, the integrand in the thermodynamic length integrals of the isothermal legs is the square root of the heat capacity, just as in the case of the iso-κ legs. We again switch to integration over $A=\frac{\kappa }{T}$, and find for the thermodynamic lengths

(83) $$L_{Q}^{\left(2\right)}=\left|\sqrt{T^{\left[2\right]}}\int_{A^{\left[2\right]}}^{A^{\left[3\right]}}\sqrt{\frac{Var_{\vec{\pi }}\left(E\left(1\right)\right)}{k_{B}}}dA\right|>L_{Q}^{\left(4\right)}=\left|\sqrt{T^{\left[1\right]}}\int_{A^{\left[4\right]}}^{A^{\left[1\right]}}\sqrt{\frac{Var_{\vec{\pi }}\left(E\left(1\right)\right)}{k_{B}}}dA\right|,$$

since $\sqrt{T^{\left[2\right]}}>\sqrt{T^{\left[1\right]}}$, $A^{\left[2\right]}=A^{\left[1\right]}$, and $A^{\left[4\right]}=A^{\left[3\right]}$ along the special adiabatic legs, and $Var_{\vec{\pi }}\left(E\left(1\right)\right)$ depends only on A.

As far as distributing the available time over the four legs, we again have to face the problem of the adiabatic legs being of zero thermodynamic length as long as we can keep the controls on target along these legs. We assume a short (i.e., $\tau_{a}\ll \tau$) time $\tau_{a}=\tau_{Q}^{\left(1\right)}=\tau_{Q}^{\left(3\right)}$ needed to move along the adiabatic leg without noticeable deviations from the ideal adiabatic curve in (κ,T) space and subtract these times from the total available time τ, $\tau_{iso-T}=\tau -2\tau_{a}$, before assigning $\tau_{Q}^{\left(2\right)}$ and $\tau_{Q}^{\left(4\right)}$ according the principle of constant thermodynamic speed. Assuming constant relaxation times, we determine the optimal times using the ratio of the thermodynamic lengths,

(84) $$\frac{\tau_{Q}^{\left(2\right)}}{\tau_{Q}^{\left(4\right)}}=\frac{L_{Q}^{\left(2\right)}}{L_{Q}^{\left(4\right)}}=\frac{\sqrt{T^{\left[2\right]}}}{\sqrt{T^{\left[1\right]}}},$$

to be $\tau_{Q}^{\left(2\right)}=\tau_{iso-T}\frac{\sqrt{T^{\left[2\right]}}}{\sqrt{T^{\left[2\right]}}+\sqrt{T^{\left[1\right]}}}$ and $\tau_{Q}^{\left(4\right)}=\tau_{iso-T}\frac{\sqrt{T^{\left[1\right]}}}{\sqrt{T^{\left[2\right]}}+\sqrt{T^{\left[1\right]}}}$. Analogously, when minimizing the entropy production instead of the excess heat, we obtain $L_{S}^{\left(2\right)}=L_{S}^{\left(4\right)}$, and thus with $\tau_{a}=\tau_{S}^{\left(1\right)}=\tau_{S}^{\left(3\right)}$, the optimal time assignment to the two legs is $\tau_{S}^{\left(2\right)}=\tau_{S}^{\left(4\right)}=\frac{\tau_{iso-T}}{2}$.

Again, due to the sum over microstates with nontrivial energy eigenvalues under the square root, further analytical calculations require additional simplifications; in particular, the optimal placement of the steps along each of the isothermal paths remains open but is expected to vary according to the specific heat.

## 5. Approximation of the Hydrogen Atom-Like System as a Two-Level System

### 5.1. Preliminaries

In many cases of statistical mechanical systems, one can find analytical solutions for a problem, if we can restrict ourselves to a two-state model; perhaps the most straightforward example for our purposes is the spin 1/2 system [27,28]. This is also the case here, since we can employ the probability $\pi_{1}$ of one of the two states as the integration variable, $\pi =\pi_{1}$. Probability conservation then determines the occupation probability of the second state as $\pi_{2}=1-\pi_{1}=1-\pi$, thus eliminating the complicated sum over the microstates. This was demonstrated in [35], where the thermodynamic length was computed for a paramagnet in a magnetic field for an isothermal path along which the energy of the paramagnet was changed by varying the magnetic field in finite time. In this fashion, one could compute the thermodynamic length and subsequently assign the steps by the equal thermodynamic length criterion, thus solving the optimal control problem.

However, the situation is more complex in the case of the cycle of the hydrogen atom-like system, because along the iso-κ legs the temperature varies, adding a nontrivial function T(π) to the integrand for the case of minimizing the excess heat. On the other hand, the approximation of any statistical mechanical system by a two-state system actually leads to many generic properties associated with the thermodynamic cycles of the two-state system, i.e., many of the results obtained below do not depend on the specific property in Equation (3), ${\tilde{E}}_{n}\left(\kappa \right)=\kappa {\tilde{E}}_{n}\left(1\right)$, of the hydrogen atom-like system.

If we take two energy levels, $n_{1}$ and $n_{2}$ with $n_{1}<n_{2}$ and thus $E_{n_{1}}\left(\kappa \right)={\tilde{E}}_{n_{1}}\left(\kappa \right)=\kappa {\tilde{E}}_{n_{1}}\left(1\right)<E_{n_{2}}\left(\kappa \right)={\tilde{E}}_{n_{2}}\left(\kappa \right)=\kappa {\tilde{E}}_{n_{2}}\left(1\right)$, with degeneracies $g\left(n_{1}\right)={\left(n_{1}\right)}^{2}$ and $g\left(n_{2}\right)={\left(n_{2}\right)}^{2}$, respectively—recall: we ignore the spin degeneracy of the electron in this study—, then we obtain the following expressions, for given (κ,T): For the probabilities, we have

(85) $$\tilde{\pi }\left(n_{1}\right)=g\left(n_{1}\right)\rho \left(n_{1}\right)=\pi =\frac{g\left(n_{1}\right)exp\left(\frac{-{\tilde{E}}_{n_{1}}\left(\kappa \right)}{k_{B}T}\right)}{g\left(n_{1}\right)exp\left(\frac{-{\tilde{E}}_{n_{1}}\left(\kappa \right)}{k_{B}T}\right)+g\left(n_{2}\right)exp\left(\frac{-{\tilde{E}}_{n_{2}}\left(\kappa \right)}{k_{B}T}\right)}$$

and

(86) $$\tilde{\pi }\left(n_{2}\right)=g\left(n_{2}\right)\rho \left(n_{2}\right)=1-\pi =\frac{g\left(n_{2}\right)exp\left(\frac{-{\tilde{E}}_{n_{2}}\left(\kappa \right)}{k_{B}T}\right)}{g\left(n_{1}\right)exp\left(\frac{-{\tilde{E}}_{n_{1}}\left(\kappa \right)}{k_{B}T}\right)+g\left(n_{2}\right)exp\left(\frac{-{\tilde{E}}_{n_{2}}\left(\kappa \right)}{k_{B}T}\right)}.$$

For the equilibrium entropy, we have the expression

(87) $$S\left(\pi \right)=-k_{B}\left[\pi ln\left(\frac{\pi }{g\left(n_{1}\right)}\right)+\left(1-\pi \right)ln\left(\frac{1-\pi }{g\left(n_{2}\right)}\right)\right]=-k_{B}\left[\pi \left(ln\left(\pi \right)-ln\left(g\left(n_{1}\right)\right)\right)+\;\left(1-\pi \right)\left(ln\left(1-\pi \right)-ln\left(g\left(n_{2}\right)\right)\right)\right]=k_{B}ln\left(Z\left(\kappa ,T\right)\right)-\pi \left(\kappa ,T\right)\frac{Bk_{B}}{T};$$

and for the temperature,

(88) $$T\left(\pi \right)=\frac{B}{ln\left[\left(\frac{g\left(n_{2}\right)}{g\left(n_{1}\right)}\right)\left(\frac{\pi }{1-\pi }\right)\right]}.$$

Here, $B=-\left({\tilde{E}}_{n_{1}}\left(\kappa \right)-{\tilde{E}}_{n_{2}}\left(\kappa \right)\right)/k_{B}>0$, for a generic two-state system; specifically for the hydrogen atom-like system, we have $B=\kappa B_{0}/k_{B}$ with $B_{0}=-\left({\tilde{E}}_{n_{1}}\left(1\right)-{\tilde{E}}_{n_{2}}\left(1\right)\right)=\alpha \left(\frac{1}{{\left(n_{1}\right)}^{2}}-\frac{1}{{\left(n_{2}\right)}^{2}}\right)>0$. Note that $\left(\frac{g\left(n_{2}\right)}{g\left(n_{1}\right)}\right)\left(\frac{\pi }{1-\pi }\right)\geq 1$, with equality only possible in the infinite temperature limit when $\pi =\frac{g\left(n_{1}\right)}{g\left(n_{1}\right)+g\left(n_{2}\right)}$. 

With this, we get for the expectation value of the energy

(89) $$\langle E\rangle \left(\kappa ,T\right)={\tilde{E}}_{n_{2}}\left(\kappa \right)+\pi \left(\kappa ,T\right)\left({\tilde{E}}_{n_{1}}\left(\kappa \right)-{\tilde{E}}_{n_{2}}\left(\kappa \right)\right)=\kappa \left[{\tilde{E}}_{n_{2}}\left(1\right)-\pi \left(\frac{\kappa }{T}\right)B_{0}\right],$$

where the second equality holds for the hydrogen atom-like system. For the derivative of the entropy with respect to π, we find

(90) $$\frac{dS}{d\pi }=-k_{B}\left[ln\left(\pi \right)-ln\left(g\left(n_{1}\right)\right)-ln\left(1-\pi \right)+ln\left(g\left(n_{2}\right)\right)+1-1\right]=\;-k_{B}ln\left(\left(\frac{g\left(n_{2}\right)}{g\left(n_{1}\right)}\right)\left(\frac{\pi }{1-\pi }\right)\right)=-k_{B}\frac{B}{T}<0$$

for all temperatures. From this follows that for all valid values of π, $\frac{g\left(n_{1}\right)}{g\left(n_{1}\right)+g\left(n_{2}\right)}\leq \pi \leq 1$, we have a one-to-one correspondence between the entropy S and the occupation probability of the low energy state π. As a consequence, every adiabatic path for any two-state system is automatically special adiabatic, because S = constant along the path implies π = constant, too.

### 5.2. Thermodynamic Cycles

With the formulas derived above, we can compute some of the expressions given in the previous section for the work and heat transfer along the legs of the cycles explicitly, such as, e.g.,

(91) $$Q^{\left(j\right)}=\int_{S^{\left[j\right]}}^{S^{\left[j+1\right]}}TdS=\int_{\pi_{in}}^{\pi_{f}}T\left(\pi \right)\frac{dS}{d\pi }d\pi =\int_{\pi_{in}}^{\pi_{f}}T\left(\pi \right)\left(-\frac{B\left(\pi \right)k_{B}}{T\left(\pi \right)}\right)d\pi =-B_{0}\int_{\pi_{in}}^{\pi_{f}}\kappa \left(\pi \right)d\pi ,$$

where the last equality only holds for the hydrogen atom-like system. Here, $\pi_{in}=\pi^{\left[j\right]}$ and $\pi_{f}=\pi^{\left[j+1\right]}$ are the values of π at the beginning and at the end of the leg (j). If we are considering an iso-κ branch, then we have $\kappa \left(\pi \right)=\kappa^{\left(j\right)}$ = constant, and thus $B\left(\pi \right)=B^{\left(j\right)}$ = constant, from which follows $Q^{\left(j\right)}=-k_{B}B^{\left(j\right)}\left(\pi_{f}-\pi_{in}\right)$. Furthermore, if in addition the temperature increases along the leg, then π(T) will decrease, $\langle E\rangle \left(\kappa^{\left(j\right)},T\right)$ will increase, and thus $Q^{\left(j\right)}>0$. We note that along such a branch, we also have ${\left(\Delta E\right)}^{\left(j\right)}=\langle E\rangle \left(\kappa^{\left(j\right)},T^{\left[j+1\right]}\right)-\langle E\rangle \left(\kappa^{\left(j\right)},T^{\left[j\right]}\right)=-k_{B}B^{\left(j\right)}\left(\pi_{f}-\pi_{in}\right)$, and thus for any two-state system, we have $Q^{\left(j\right)}={\left(\Delta E\right)}^{\left(j\right)}$ and $W^{\left(j\right)}=0$ along the iso-κ branches. This is a special case of the earlier general result, Equation (19).

For the adiabatic path (j), we recall that these paths are special for any two-state system, i.e., π = constant, and thus the heat term yields $Q^{\left(j\right)}=-\int_{\pi_{in}}^{\pi_{f}}k_{B}B\left(\pi \right)d\pi =0$ since $\pi_{f}=\pi_{in}$. Finally, for an isothermal leg, we have

(92) $$Q^{\left(j\right)}=-k_{B}T^{\left(j\right)}\left[\left(\pi_{f}ln\left(\frac{\pi_{f}}{g\left(n_{1}\right)}\right)+\left(1-\pi_{f}\right)ln\left(\frac{1-\pi_{f}}{g\left(n_{2}\right)}\right)\right)-\left(\pi_{in}ln\left(\frac{\pi_{in}}{g\left(n_{1}\right)}\right)+\left(1-\;\pi_{in}\right)ln\left(\frac{1-\pi_{in}}{g\left(n_{2}\right)}\right)\right)\right]=k_{B}T^{\left(j\right)}ln\left[\frac{Z\left(\kappa^{\left[j+1\right]}/T^{\left(j\right)}\right)}{Z\left(\kappa^{\left[j\right]}/T^{\left(j\right)}\right)}\right]+B_{0}\left[\pi_{in}\kappa^{\left[j\right]}-\pi_{f}\kappa^{\left[j+1\right]}\right],$$

where the last equality holds for the hydrogen atom-like system.

In the following, we are not going to re-compute for the two-state hydrogen atom-like system all the general work and heat-related expressions derived in Section 4. We only note that for the cycles with iso-κ legs, we find again that $\eta_{\left\{\kappa ;T\right\}}=1$ and $\eta_{\left\{\kappa ;S\right\}}=1$, which are the same results as we had obtained for the complete hydrogen atom-like system, Equations (62) and (70). Thus, for the two-state system, the net work performed by the “atom” along the isothermal or adiabatic legs equals the net heat added to the system over the whole cycle because $Q^{\left(j\right)}={\left(\Delta E\right)}^{\left(j\right)}$ along the iso-κ legs. Finally, for the adiabatic-isothermal path, we find $\eta_{\left\{S;T\right\}}^{adiabatic}={\left\{1+Y\right\}}^{-1}$ and $\eta_{\left\{S;T\right\}}^{isothermal}={\left\{1+\frac{1}{Y}\right\}}^{-1}$, where $Y=\frac{k_{B}\left(T_{1}-T_{2}\right)ln\left[\frac{Z\left(\kappa_{in}/T_{1}\right)}{Z\left(\kappa_{f}/T_{2}\right)}\right]}{\kappa_{f}\left(1-\frac{T_{1}}{T_{2}}\right)\left\{{\tilde{E}}_{n_{2}}\left(1\right)\left[1-\left(\frac{\kappa_{in}/T_{1}}{\kappa_{f}/T_{2}}\right)\right]-B_{0}\left[\pi \left(\kappa_{f}/T_{2}\right)-\left(\frac{\kappa_{in}/T_{1}}{\kappa_{f}/T_{2}}\right)\pi \left(\kappa_{in}/T_{1}\right)\right]\right\}}$.

Instead, we will focus on the optimal control problem of minimizing the excess heat production instead. Applying the general formulas in Equations (23) and (24), we observe that the expression for the square length of an infinitesimal distance in probability space yields in terms of π:

(93) $${\left(dL_{Q}\right)}^{2}=k_{B}T\left(\pi \right)\left(\begin{array}{c} d\pi \\ -d\pi \end{array}\right)\left\{\begin{array}{cc} \left(\frac{g\left(n_{1}\right)}{\pi }\right)\frac{g\left(n_{1}\right)}{{\left(g\left(n_{1}\right)\right)}^{2}} & 0\\ 0 & \left(\frac{g\left(n_{2}\right)}{1-\pi }\right)\frac{g\left(n_{2}\right)}{{\left(g\left(n_{2}\right)\right)}^{2}} \end{array}\right\}\left(\begin{array}{c} d\pi \\ -d\pi \end{array}\right)\;=k_{B}T\left(\pi \right)\left[{\left(d\pi \right)}^{2}\right]\left(\frac{1}{\pi }+\frac{1}{1-\pi }\right).$$

The metric for the energy levels is a compactified (2×2) version of the true $\left(g\left(n_{1}\right)+g\left(n_{2}\right)\right)\times \left(g\left(n_{1}\right)+g\left(n_{2}\right)\right)$ microstate metric ${\left\{\frac{1}{\overset{↔}{\pi }}\right\}}_{kl}=\delta_{kl}\frac{1}{r_{1}}=\delta_{kl}\frac{g\left(n_{1}\right)}{\pi }$ for $k=1,\ldots ,g\left(n_{1}\right)$ and ${\left\{\frac{1}{\overset{↔}{\pi }}\right\}}_{kl}=\delta_{kl}\frac{1}{r_{2}}=\delta_{kl}\frac{g\left(n_{2}\right)}{1-\pi }$ for $k=g\left(n_{1}\right)+1,\ldots ,g\left(n_{1}\right)+g\left(n_{2}\right)$. Similarly, $\left(\begin{array}{c} d\pi \\ -d\pi \end{array}\right)$ is a compactified version of the $\left(g\left(n_{1}\right)+g\left(n_{2}\right)\right)$-dimensional microstate probability difference vector $\left(d\rho_{1},\ldots ,d\rho_{1},-d\rho_{2},\ldots ,-d\rho_{2}\right)=\left(\frac{d\pi }{g\left(n_{1}\right)},\ldots ,\frac{d\pi }{g\left(n_{1}\right)},\frac{-d\pi }{g\left(n_{2}\right)},\ldots ,\frac{-d\pi }{g\left(n_{2}\right)}\right)$. As mentioned earlier, we assume that the microstates with the same energy exhibit the same occupation probability, and that we do not have “mixing” among the states for the same energy, i.e., all $g\left(n_{1}\right)$ states with energy “gain” the same amount of probability $d\rho_{1}=\frac{d\pi }{g\left(n_{1}\right)}$, and similarly, all states with energy “lose” the same amount of probability.

Taking this into account, we find for the infinitesimal element of thermodynamic length:

(94) $$dL_{Q}=\sqrt{k_{B}T\left(\pi \right)}\sqrt{\frac{1}{\pi }+\frac{1}{1-\pi }}\left|d\pi \right|=\pm \sqrt{\frac{k_{B}T\left(\pi \right)}{\pi \left(1-\pi \right)}}d\pi ,$$

where the (+)-sign is employed if π increases along the path (dπ>0) and the (−)-sign is employed if π decreases along the path (dπ<0). We note that Equations (93) and (94) apply to any two-state system and are not restricted to hydrogen atom-like systems. As mentioned above, the adiabatic pieces of the cycle are special for a two-state system, and thus there is no excess heat production to first order since π is constant along these legs, and thus |dπ|=0; alternatively, we can note that the initial and final values of π, $\pi_{in}$, and $\;\pi_{f}$, respectively, for an adiabatic path are the same, $\pi_{f}=\pi_{in}$, and thus $\pm \int_{\pi_{in}}^{\pi_{f}}\frac{\sqrt{k_{B}T\left(\pi \right)}}{\sqrt{\pi \left(1-\pi \right)}}d\pi =\pm \int_{\pi_{in}}^{\pi_{in}}\frac{\sqrt{k_{B}T\left(\pi \right)}}{\sqrt{\pi \left(1-\pi \right)}}d\pi =0$. However, we should introduce a phenomenological time $\tau_{a}$ required for the external controls to follow the ideal path along an adiabatic leg. 

For the isothermal pieces of the cycle, we can compute the corresponding thermodynamic lengths $L_{Q}$ analytically, since

(95) $$\pm \int_{\pi_{in}}^{\pi_{f}}\frac{d\pi }{\sqrt{\pi \left(1-\pi \right)}}=\pm \left(arcsin\left(1-2\pi_{in}\right)-arcsin\left(1-2\pi_{f}\right)\right).$$

Assuming constant relaxation times ${\left(\Delta t\right)}_{T}$ along the path, the optimal assignment of time follows automatically; along the optimal path with optimal times $\tau^{\left(2\right)}$ and $\tau^{\left(4\right)}$, and thus $N^{\left(2\right)}=\frac{\tau^{\left(2\right)}}{{\left(\Delta t\right)}_{T}}$ and $N^{\left(4\right)}=\frac{\tau^{\left(4\right)}}{{\left(\Delta t\right)}_{T}}$ given, we find

(96) $$\pi_{i}^{\left(2\right)}=\frac{1}{2}\left[1-sin\left(arcsin\left(1-2\pi_{in}^{\left(2\right)}\right)-\left[arcsin\left(1-2\pi_{in}^{\left(2\right)}\right)-arcsin\left(1-2\pi_{f}^{\left(2\right)}\right)\right]\frac{i}{N^{\left(2\right)}}\right)\right]$$

along leg 2, and

(97) $$\pi_{i}^{\left(4\right)}=\frac{1}{2}\left[1-sin\left(arcsin\left(1-2\pi_{f}^{\left(4\right)}\right)-\left[arcsin\left(1-2\pi_{f}^{\left(4\right)}\right)-arcsin\left(1-2\pi_{in}^{\left(4\right)}\right)\right]\frac{N^{\left(4\right)}-i}{N^{\left(4\right)}}\right)\right]=\;\frac{1}{2}\left[1-sin\left(arcsin\left(1-2\pi_{in}^{\left(4\right)}\right)+\left[arcsin\left(1-2\pi_{f}^{\left(4\right)}\right)-arcsin\left(1-2\pi_{in}^{\left(4\right)}\right)\right]\frac{i}{N^{\left(4\right)}}\right)\right]$$

along leg 4. Note that we have employed the (+)-sign in the integral for leg 2 since $\pi_{f}^{\left(2\right)}>\pi_{in}^{\left(2\right)}$, and similarly the (−)-sign for leg 4 since $\pi_{in}^{\left(4\right)}>\pi_{f}^{\left(4\right)}$.

However, for the iso-κ leg, we need to compute the integral

(98) $$\left(\pm \right)\int_{\pi_{in}}^{\pi_{f}}\frac{\sqrt{Bk_{B}}d\pi }{\sqrt{\left(\pi \left(1-\pi \right)\right)ln\left[\left(\frac{g\left(n_{2}\right)}{g\left(n_{1}\right)}\right)\left(\frac{\pi }{1-\pi }\right)\right]}}=\left(\pm \right)\sqrt{\kappa }\int_{\pi_{in}}^{\pi_{f}}\frac{\sqrt{B_{0}}d\pi }{\sqrt{\left(\pi \left(1-\pi \right)\right)ln\left[\left(\frac{g\left(n_{2}\right)}{g\left(n_{1}\right)}\right)\left(\frac{\pi }{1-\pi }\right)\right]}},$$

which is not straightforward even for constant B. Here, the (+)-sign refers to legs with increasing π (i.e., leg 3) and the (−)-sign to legs with decreasing π (i.e., leg 1), and we have used Equation (88) to replace T(π). Thus, any cycle containing an iso-κ leg does not allow us to compute the actual distribution of steps along the leg once the optimal time assignment to the legs has been performed. Note that the $\sqrt{\kappa }$ dependence of the thermodynamic length of an iso-κ branch is specific to the hydrogen atom-like system and is not a property of a general two-state system. 

Nevertheless, we note that for an iso-κ-adiabatic cycle, $\pi^{\left[2\right]}=\pi^{\left[3\right]}$ and $\pi^{\left[1\right]}=\pi^{\left[4\right]}$ because of the special adiabaticity of legs (2) and (4), where we assume as usual that we need the time $\tau_{a}$ to proceed along an adiabatic leg without generating excess heat such that the available time is $\tau_{iso-\kappa }=\tau -2\tau_{a}$. Then we determine the optimal assignment of times as $\tau_{Q}^{\left(1\right)}=\tau_{iso-\kappa }\frac{\sqrt{\kappa_{in}}}{\sqrt{\kappa_{in}}+\sqrt{\kappa_{f}}}$ and $\tau_{Q}^{\left(3\right)}=\tau_{iso-\kappa }\frac{\sqrt{\kappa_{f}}}{\sqrt{\kappa_{in}}+\sqrt{\kappa_{f}}}$, according to the ratio of the optimal times, $\frac{\tau_{Q}^{\left(3\right)}}{\tau_{Q}^{\left(1\right)}}=\frac{L_{Q}^{\left(3\right)}}{L_{Q}^{\left(1\right)}}=\frac{\sqrt{\kappa_{f}}}{\sqrt{\kappa_{in}}}$; we had found the same result for the general hydrogen atom-like system in Equation (66). In contrast to this result, for the iso-κ-isothermal cycle, the relative sizes of the four legs are unknown, and thus we cannot assign optimal times to the four legs of this cycle, even though we have an analytical expression for the thermodynamic lengths of the two isothermal legs.

Among the three cycles we have considered, only the isothermal-adiabatic one can be completely solved analytically when minimizing excess heat, yielding $L_{Q}^{\left(1\right)}=L_{Q}^{\left(3\right)}=0$ and

(99) $$L_{Q}^{\left(2\right)}=\sqrt{k_{B}T^{\left[2\right]}}\left(arcsin\left(1-2\pi^{\left[2\right]}\right)-arcsin\left(1-2\pi^{\left[3\right]}\right)\right)$$

and

(100) $$L_{Q}^{\left(4\right)}=\sqrt{k_{B}T^{\left[1\right]}}\left(arcsin\left(1-2\pi^{\left[1\right]}\right)-arcsin\left(1-2\pi^{\left[4\right]}\right)\right).$$

Due to the special adiabaticity of legs 1 and 3, we have $\pi^{\left[1\right]}=\pi^{\left[2\right]}$ and $\pi^{\left[3\right]}=\pi^{\left[4\right]}$, and thus

(101) $$L_{Q}^{\left(4\right)}=\sqrt{k_{B}T^{\left[1\right]}}\left(arcsin\left(1-2\pi^{\left[2\right]}\right)-arcsin\left(1-2\pi^{\left[3\right]}\right)\right)=\sqrt{\frac{T^{\left[1\right]}}{T^{\left[2\right]}}}L_{Q}^{\left(2\right)}.$$

Subtracting the time needed for the adiabatic legs, we can split the remaining time $\tau_{iso-T}=\tau -2\tau_{a}$ over the legs 2 and 4, i.e.,

(102) $$\tau_{Q}^{\left(2\right)}=\tau_{iso-T}\frac{L_{Q}^{\left(2\right)}}{L_{cycle}}=\tau_{iso-T}\frac{\sqrt{T^{\left[2\right]}}}{\sqrt{T^{\left[1\right]}}+\sqrt{T^{\left[2\right]}}}$$

and

(103) $$\tau_{Q}^{\left(4\right)}=\tau_{iso-T}\frac{L_{Q}^{\left(4\right)}}{L_{cycle}}=\tau_{iso-T}\frac{\sqrt{T^{\left[1\right]}}}{\sqrt{T^{\left[1\right]}}+\sqrt{T^{\left[2\right]}}}.$$

The ratio of the two assigned times is the same as that which we had found in Equation (84),

(104) $$\frac{\tau_{Q}^{\left(2\right)}}{\tau_{Q}^{\left(4\right)}}=\frac{L_{Q}^{\left(2\right)}}{L_{Q}^{\left(4\right)}}=\sqrt{\frac{T^{\left[2\right]}}{T^{\left[1\right]}}},$$

i.e., we need to spend more time in the high-temperature leg than in the low-temperature leg when minimizing the excess heat of the system. The assignment of the step points along each leg now follows directly from Equations (96) and (97).

We note that we did not need to employ the special properties of the hydrogen atom-like system to derive the finite-time thermodynamics results in Equations (95)–(97) and (99)–(104). Thus, the optimal ratio of assigned times $\sqrt{T^{\left[2\right]}/T^{\left[1\right]}}$ is the optimality criterion for the adiabatic-isothermal cycle of any two-state system. This agrees with the analysis of a spin 1/2 two-state system in finite time where the power along the cycle was maximized [28], and is reminiscent of similar general results obtained in the analysis of Carnot cycles in finite time [10]. 

The above analysis was performed using the metric ${\overset{↔}{M}}_{Q}$ in the optimality criterion for minimizing excess heat/work. If we employ the metric ${\overset{↔}{M}}_{S}$ appropriate for minimizing entropy production, we note that we now have $dL_{S}=\sqrt{k_{B}}\sqrt{\frac{1}{\pi }+\frac{1}{1-\pi }}\left|d\pi \right|=\pm \sqrt{\frac{k_{B}}{\pi \left(1-\pi \right)}}d\pi$. Thus, for every leg (j), we can use Equation (95) to compute the thermodynamic length $L_{S}^{\left(j\right)}$, regardless of whether we are dealing with an adiabatic, isothermal, or iso-κ leg. As a consequence, all three cycles considered can be solved analytically. For the Carnot-like cycle, we obtain an assignment of equal times to the isothermal legs,$\;\tau_{S}^{\left(2\right)}=\tau_{S}^{\left(4\right)}$, like we had obtained for the full hydrogen atom; this assignment is again analogous to results obtained for minimal entropy production in the spin 1/2 two-state model [28], as one would have expected from the universal aspects of two-level systems. For the Otto-like cycle, we also find equal time assignments for the iso-κ legs, $\tau_{S}^{\left(1\right)}=\tau_{S}^{\left(3\right)}$, as we did already for the full hydrogen atom. Finally, for the Stirling-like cycle, the total thermodynamic length is $L_{S;cycle}=2\left[arcsin\left(1-2\pi^{\left[2\right]}\right)-arcsin\left(1-2\pi^{\left[4\right]}\right)\right]$, and thus the optimal time assignment to the four legs is $\tau_{S}^{\left(1\right)}=\frac{\tau }{2}\frac{\left[arcsin\left(1-2\pi^{\left[2\right]}\right)-arcsin\left(1-2\pi^{\left[1\right]}\right)\right]}{\left[arcsin\left(1-2\pi^{\left[2\right]}\right)-arcsin\left(1-2\pi^{\left[4\right]}\right)\right]}$, $\tau_{S}^{\left(2\right)}=\frac{\tau }{2}\frac{\left[arcsin\left(1-2\pi^{\left[2\right]}\right)-arcsin\left(1-2\pi^{\left[3\right]}\right)\right]}{\left[arcsin\left(1-2\pi^{\left[2\right]}\right)-arcsin\left(1-2\pi^{\left[4\right]}\right)\right]}$, $\tau_{S}^{\left(3\right)}=\frac{\tau }{2}\frac{\left[arcsin\left(1-2\pi^{\left[3\right]}\right)-arcsin\left(1-2\pi^{\left[4\right]}\right)\right]}{\left[arcsin\left(1-2\pi^{\left[2\right]}\right)-arcsin\left(1-2\pi^{\left[4\right]}\right)\right]}$ and $\tau_{S}^{\left(4\right)}=\frac{\tau }{2}\frac{\left[arcsin\left(1-2\pi^{\left[1\right]}\right)-arcsin\left(1-2\pi^{\left[4\right]}\right)\right]}{\left[arcsin\left(1-2\pi^{\left[2\right]}\right)-arcsin\left(1-2\pi^{\left[4\right]}\right)\right]}$. Since we have analytical expressions for the thermodynamic length for every leg, it is straightforward to also assign the times along the legs for all three cycles, analogous to Equations (96) and (97). 

## 6. Summary and Discussion

In the previous sections, we have presented three thermodynamic cycles for a hydrogen atom-like system in (κ,T) space, where κ allows us to control the electronic energy levels of the system: iso-κ-isothermal, iso-κ-adiabatic, and adiabatic-isothermal. We have written down expressions for heat and work along the legs of these cycles and derived conditions that yield optimal ways to run through the cycles in finite time such that the entropy production or the excess heat production is minimal. In particular, we found that the optimal allocation of time—in units of relaxation-to-equilibrium times—including the allocation of discrete steps along the cycle, should take place in such a way that a) the path in (κ,T) space, for given corners ([1],[2],[3],[4]) and prescribed types of branches, should be chosen such that the total thermodynamic length of the cycle is a minimum, b) the time allocated to each leg of the cycle should be proportional to the thermodynamic length of each leg separately, and c) the discrete steps along each leg should be spaced in such a way that the thermodynamic lengths between all pairs of consecutive points along the branch are identical. We showed that the thermodynamic length could be evaluated using an appropriate metric, ${\overset{↔}{M}}_{Q}=\left\{\frac{k_{B}T_{i}}{{\overset{↔}{\pi }}_{i}}\right\}$ or ${\overset{↔}{M}}_{S}=\left\{\frac{k_{B}}{{\overset{↔}{\pi }}_{i}}\right\}$, in probability distribution space, which was obtained as part of the optimal control analysis.

We note that condition a) is trivial for the cycles chosen in the present study, since each leg is completely determined by the assignment of its end points and the type of path, i.e., whether it is iso-κ, isothermal, or adiabatic. If there were several control parameters $\kappa_{a,b,\ldots }$ that influence the change in the energy levels of the system, then step a) would be a major part of the optimal control problem of minimizing entropy or excess heat production, of course.

Furthermore, we discussed the minimization of excess heat along special adiabatic branches, which by construction equals zero due to the condition that the equilibrium probability distribution is constant along such a special adiabatic path, and thus never an imbalance between actual and equilibrium distribution can build up; for example, by construction, all two-state statistical mechanical systems constitute examples where adiabatic paths are special. Thus, only our inability in practice to keep the control parameters on-target while moving along a special adiabatic path generates deviations from the equilibrium occupation of the microstates of the system, which are the main source of excess heat or entropy production. We derived approximate expressions for the thermodynamic length of generic off-target paths that still remain close to the ideal adiabatic path.

We note that the issue of on-target path control arises for every leg of any cycle, but that one usually ignores these contributions to the entropy or excess heat production. The reason for discounting them is twofold; for one, they tend to be overwhelmed by the effects of having only finite time available to run through the legs of the cycle. Perhaps more important is the fact that an analysis would require information about the apparatus employed to move the system in the (κ,T) space, which is specific to each experiment, and thus usually not within the purview of the theoretical study. 

Related finite-time analyses have been performed for thermodynamic engines in the past by assuming, e.g., a generic heat leakage or (inefficient) heat conduction during the processes of the cycle, which are described by phenomenological laws [43,44,45]. However, this leakage was not connected to the issue of being “on-target” vs. “off-target”; instead, the thermodynamic controls were assumed to be perfect, and the inefficiencies associated with, e.g., friction or heat conduction were considered part of the working of the engine.

These optimality criteria and the associated thermodynamic metrics are very general and apply to essentially all statistical mechanical systems, as long as the energy levels can be controlled by a generic parameter κ or set of parameters $\vec{\kappa }=\left(\kappa_{a},\kappa_{b},\ldots \right)$. Thus, there are connections to other general results [35,46,47,48,49,50]. For example, the excess heat we consider corresponds to the excess work investigated by Sivak and Crooks [47], and thus Equations (28) and (29) for the thermodynamic lengths $L_{Q/S}$ correspond to the generalized thermodynamic length they define via the time-integrated force covariance matrix [47]. Furthermore, the relationship ${\overset{↔}{M}}_{Q}=T{\overset{↔}{M}}_{S}$ for statistical mechanical systems observed in this study corresponds to the conformal equivalence of the energy and entropy metric demonstrated by Salamon and co-workers for thermodynamic equilibrium systems [50]. This also shows that the excess heat metric ${\overset{↔}{M}}_{Q}$ measures the dissipation or loss of availability when proceeding along the path in finite time, as had been shown earlier in the context of optimally measuring free energy differences in statistical mechanical systems [35] and computing thermodynamic lengths within computer simulations [46]. 

Specific to the hydrogen atom-like system is the observation that $E_{n}\left(\kappa \right)=\kappa E_{n}\left(\kappa =1\right)$ for all energy levels n. From this follow certain simplifications, such as the fact that κ and T appear only in the combination $A=\frac{\kappa }{T}$ in the equilibrium probability distribution $\vec{\pi }$, and therefore all adiabatic paths in (κ,T) space are special and lie on straight lines that contain the origin (κ=0,T=0). In particular, we observed that for the isothermal-adiabatic cycle the time allocation to the two isothermal branches should be proportional to the square roots of the temperatures associated with these branches when minimizing the excess heat production. Similarly, for the iso-κ-adiabatic cycle, the time allocation for the two iso-κ branches should be proportional to the square roots of the κ values associated with these branches. In contrast, when minimizing the entropy production of the cycles that contain two adiabatic branches, the optimal times assigned to the two isothermal or iso-κ-legs should be equal. Here, we note that the results obtained in Section 4 would be applicable to any system whose energy spectrum scales with the control parameter κ according to Equation (3), such as, e.g., a quantum harmonic oscillator if one modifies only the basic frequency, $\omega_{0}\to \kappa \omega_{0}$, or a spin system with $N_{L}$ energy levels in a magnetic field as long as we only change the strength of the applied magnetic field, $B_{0}\to \kappa B_{0}$. For an analysis of the influence of the eigenvalue spectrum of a system on the finite-time performance, we refer to [51].

For the case of the hydrogen atom-like system, we have also considered a two-state approximation, which allows us to perform further analytical evaluations of the optimal control conditions. When minimizing excess heat, we can analytically solve the isothermal-adiabatic cycle, while for minimal entropy production, all three cycles can now be solved analytically. These results are quite general and hold for any two-state system, as can be seen from the agreement with results obtained from, e.g., a spin-1/2 system [28]. Furthermore, the result for the Carnot cycle is reminiscent to the outcome of some finite time optimal control calculations for heat engines, working between two reservoirs [10], and agrees with corresponding results for the spin 1/2 system [28].

However, thermal interactions with the environment affect all energy levels, making the two-state approximation of the hydrogen atom somewhat artificial. On the other hand, enforcing transitions via narrow band radiation allows us to focus on single pairs of energy levels, thus providing a more realistic example of a two-state system at the price of dealing with an a-thermal cycle.

**Figure 5:**
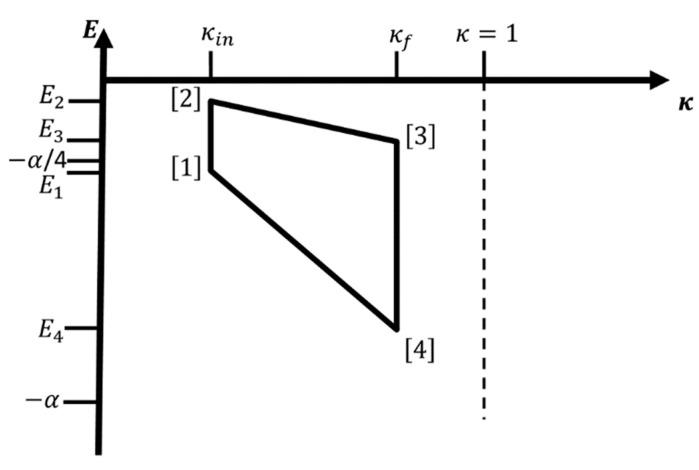
Sketch of an a-thermal iso-κ-“adiabatic” cycle for a two-state system comprising the ground state and the first excited state of the hydrogen-like atom. Branches [1]→[2] and [3]→[4] are iso-κ-legs and branches [2]→[3] and [4]→[1] are “adiabatic” legs, respectively. Note that the adiabatic legs run along straight lines that pass through the origin and through the point (κ,E)=(1,−α) for the adiabatic leg in the ground state, and through the origin and through the point (κ,E)=(1,−α/4) for the adiabatic leg in the first excited state, respectively. The four corners of the cycle are the points $\left[1\right]=\left(\kappa^{\left[1\right]},E^{\left[1\right]}\right)=\left(\kappa_{in},E_{1}\right)$, $\left[2\right]=\left(\kappa^{\left[2\right]},E^{\left[2\right]}\right)=\left(\kappa_{in},E_{2}\right)$, $\left[3\right]=\left(\kappa^{\left[3\right]},E^{\left[3\right]}\right)=\left(\kappa_{f},E_{3}\right)$, and $\left[4\right]=\left(\kappa^{\left[4\right]},E^{\left[4\right]}\right)=\left(\kappa_{f},E_{4}\right)$ in the (κ,E) plane. Note that $E^{\left[1\right]}=\kappa_{in}E_{1}\left(\kappa =1\right)$, $E^{\left[2\right]}=\kappa_{in}E_{2}\left(\kappa =1\right)$, $E^{\left[3\right]}=\kappa_{f}E_{2}\left(\kappa =1\right)$, and $E^{\left[4\right]}=\kappa_{f}E_{1}\left(\kappa =1\right)$.

A possible four-leg cycle for the two-state version of a single hydrogen atom-like system (i.e., only two of the electronic energy levels $n_{1}$ and $n_{2}>n_{1}$ participate in the process) without contact to a heat bath is shown in Figure 5 for the case $n_{1}=1$ and $n_{2}=2$. No temperature is involved, and the cycle runs as follows, where we take as starting point the atom in the state $n_{1}$ with energy $E^{\left[1\right]}=E_{n_{1}}\left(\kappa_{in}\right)$ for $\kappa^{\left[1\right]}=\kappa_{in}$. In leg 1, we excite the atom from $n=n_{1}$ to $n=n_{2}$ via irradiation at frequency $\nu_{in}$ ($h\nu_{in}=E^{\left[2\right]}-E^{\left[1\right]}=E_{n_{2}}\left(\kappa_{in}\right)-E_{n_{1}}\left(\kappa_{in}\right)$), while we keep κ at the value $\kappa_{in}$,$\;\kappa^{\left[2\right]}=\kappa_{in}$. Next, we increase κ to $\kappa^{\left[3\right]}=\kappa_{f}$, while keeping the atom in the excited state $n=n_{2}$, i.e., $E^{\left[3\right]}=E_{n_{2}}\left(\kappa_{f}\right)$. This is followed by the reverse operations, i.e., we de-excite the atom back to $n\;=\;n_{1}$ via irradiation at frequency $\nu_{f}$ ($h\nu_{f}=E^{\left[3\right]}-E^{\left[4\right]}=E_{n_{2}}\left(\kappa_{f}\right)-E_{n_{1}}\left(\kappa_{f}\right)$), while keeping κ at the value $\kappa_{f}$,$\;\kappa^{\left[4\right]}=\kappa_{f}$, followed by the decrease of κ to $\kappa =\kappa_{in}$, while keeping the atom in the state $n\;=\;n_{1}$, thus closing the cycle.

Note that legs 2 and 4 would be the analogues to adiabatic branches in the thermal cycle. There, we perform or extract work on the system by changing the energy content of the hydrogen atom-like system from $E_{n_{2}}\left(\kappa_{in}\right)$ to $E_{n_{2}}\left(\kappa_{f}\right)$ and from $E_{n_{1}}\left(\kappa_{f}\right)$ to $E_{n_{1}}\left(\kappa_{in}\right)$, respectively. The total amount of work (done by the atom) associated with these two legs would be

(105) $$W=-\Delta E=-\left[E_{n_{2}}\left(\kappa_{f}\right)-E_{n_{2}}\left(\kappa_{in}\right)\right]-\left[E_{n_{1}}\left(\kappa_{in}\right)-E_{n_{1}}\left(\kappa_{f}\right)\right]=\left(\kappa_{in}-\kappa_{f}\right)\left[E_{n_{2}}\left(\kappa =1\right)-\;E_{n_{1}}\left(\kappa =1\right)\right]=\alpha \left(\frac{1}{{\left(n_{1}\right)}^{2}}-\frac{1}{{\left(n_{2}\right)}^{2}}\right)\left(\kappa_{in}-\kappa_{f}\right)<0,$$

since $\kappa_{f}>\kappa_{in}$ and $n_{2}>n_{1}$. Thus, our external apparatus, which changes κ, performs a net amount of work on the atom along these two legs.

Concerning legs 1 and 3, which would be the analogues to the heating and cooling branches of a typical thermodynamic cycle, it is not obvious how to account for the equivalent of heat transferred from and to the heat reservoir at different temperatures, and thus make a connection to thermodynamic cycles. If the radiation fields had a black-body frequency distribution, the formulas of radiation thermodynamics would apply [52,53,54], but this would weaken the desired approximation of the system through only two energy levels. What we can consider is a “reservoir” of photons with frequencies $\nu_{in}$ and $\nu_{f}$, which the hydrogen atom-like system is in contact with during the excitation and de-excitation processes in legs 1 and 3, respectively. However, such a radiation field that consists of narrow frequency bands does not act as a standard thermal heat source as far as the hydrogen atom-like system is concerned. Instead, we treat the radiation field as part of the external apparatus for the purpose of this discussion, analogous to the pressure we might apply to the material to change the properties of the excitons representing the hydrogen atom-like system. As a consequence, the net work done by the radiation field on the atom equals

(106) $$h\nu_{in}-h\nu_{f}=\left[E_{n_{2}}\left(\kappa_{in}\right)-E_{n_{1}}\left(\kappa_{in}\right)\right]-\left[E_{n_{2}}\left(\kappa_{f}\right)-E_{n_{1}}\left(\kappa_{f}\right)\right]=\left(\kappa_{in}-\kappa_{f}\right)\left[E_{n_{2}}\left(1\right)-E_{n_{1}}\left(1\right)\right]<0,$$

i.e., the energy of the radiation field shows a net increase along legs 1 and 3 by the same amount as the atom gained in energy when we applied work along legs 2 and 4. 

We can thus visualize the two-state approximation of our hydrogen atom-like system as an “engine” that acts as a “frequency conversion pump” while keeping the number of photons conserved. A possible co-efficient of performance would be

(107) $$\eta^{conv}=\frac{W^{\left(1\right)}+W^{\left(3\right)}}{W^{\left(2\right)}+W^{\left(4\right)}}=1,$$

i.e., the a-thermal cycle of the single controllable hydrogen atom-like system represents a perfect energy conversion apparatus from photons with one frequency to photons with another frequency. We note that this perfect restriction of the hydrogen atom-like system to a two-state system would also work if the modification of the Hamiltonian breaks the symmetry of the Coulomb field, as might be the case by exposing the hydrogen atom to an anisotropic external electric field inside a cavity.

Of course, in practice, this a-thermal cycle will encounter finite-time losses, too. The sources of possible finite-time losses have to be identified in an analysis of the quantum processes involved when changing the Hamiltonian and exciting/de-exciting the electrons between the two levels, including the degree of coherence maintained in the system along each leg. An analysis of these issues would go beyond the purview of this study, however. Possible approaches on how to analyze these issues can be found in some of the cited references dealing explicitly with the quantum aspects of heat engines [18,19,20,21,22,23,24,25,26,27,28,29,48,49,51]. 

A final question is whether there are experimental systems where the thermodynamic or a-thermal cycles discussed in this study might be realized. The most straightforward suggestion would be to take an individual hydrogen atom inside a cavity whose shape can be changed, such that the energy levels can be modified in some way [31]. This system should allow an a-thermal cycle, but the momentum transfer during absorption would start injecting kinetic energy, which would re-appear as a source of heat to the system. In the case of a thermodynamic cycle, where we were to expose the atom to a heat reservoir over a sufficiently large temperature range for exciting the hydrogen atom(s) to a noticeable degree, the system’s translational degrees of freedom would quickly establish thermal movement with all that implies, possibly overwhelming the thermodynamic quantities of interest in the electronic two-state system. However, since the experimental control of the hydrogen atom as a thermal engine based on the translational degrees of freedom has been quite successful already [13], a quasi-a-thermal or a thermodynamic engine based on the occupation of the electronic states and on the modification of their energy levels should be feasible. 

An alternative would be an excitonic defect in a solid, since this is usually more localized (but still mobile, in principle) and thus probably less susceptible to the kinetic thermal contributions. Furthermore, the reference value of κ, $\kappa_{in}$, should be rather small, and thus small changes in temperature would already lead to noticeable changes in the occupation probabilities of the excitonic states. On the other hand, as mentioned earlier, varying κ in a solid can be nontrivial, since the quantities entering κ will depend on both pressure—which would be a suitable control variable—and temperature. As a consequence, in a (p,T) space representation, the iso-κ branches would involve closely coordinated changes both in pressure and temperature. Additionally, an a-thermal cycle might be difficult to achieve, due to the strong thermal coupling of the exciton to the rest of the solid. Nevertheless, it appears that a variety of systems exist which might serve as substrates for the realization of an electronic states-based engine with a single hydrogen atom-like system as the working fluid.
